# Distortion of the bilayer and dynamics of the BAM complex in lipid nanodiscs

**DOI:** 10.1038/s42003-020-01419-w

**Published:** 2020-12-14

**Authors:** Matthew G. Iadanza, Bob Schiffrin, Paul White, Matthew A. Watson, Jim E. Horne, Anna J. Higgins, Antonio N. Calabrese, David J. Brockwell, Roman Tuma, Antreas C. Kalli, Sheena E. Radford, Neil A. Ranson

**Affiliations:** 1 Astbury Centre for Structural Molecular Biology, School of Molecular & Cellular Biology, Faculty of Biological Sciences, University of Leeds, Leeds, LS2 9JT UK; 2 Faculty of Science, University of South Bohemia, Ceske Budejovice, Czech Republic

**Keywords:** Cryoelectron microscopy, Protein folding

## Abstract

The β-barrel assembly machinery (BAM) catalyses the folding and insertion of β-barrel outer membrane proteins (OMPs) into the outer membranes of Gram-negative bacteria by mechanisms that remain unclear. Here, we present an ensemble of cryoEM structures of the *E. coli* BamABCDE (BAM) complex in lipid nanodiscs, determined using multi-body refinement techniques. These structures, supported by single-molecule FRET measurements, describe a range of motions in the BAM complex, mostly localised within the periplasmic region of the major subunit BamA. The β-barrel domain of BamA is in a ‘lateral open’ conformation in all of the determined structures, suggesting that this is the most energetically favourable species in this bilayer. Strikingly, the BAM-containing lipid nanodisc is deformed, especially around BAM’s lateral gate. This distortion is also captured in molecular dynamics simulations, and provides direct structural evidence for the lipid ‘disruptase’ activity of BAM, suggested to be an important part of its functional mechanism.

## Abstract (web summary)

With cryo-EM, single-molecule FRET and MD simulations, Iadanza et al. characterise the membrane protein insertase complex BAM in lipid bilayer nanodiscs. They show that the β-barrel domain of BamA is in a ‘lateral open’ conformation, and that BAM-containing lipid nanodisc deform around BAM’s lateral gate, giving structural evidence for lipid ‘disruptase’ activity of BAM.

## Introduction

The β-barrel assembly machinery (BAM complex), is a molecular machine in the outer membranes (OM) of Gram-negative bacteria that is essential for the folding and insertion of β-barrel OM proteins (OMPs) into the OM^1–3^. BAM’s known substrates include OMPs containing between 8 and 26 β-strands^4^, and BAM is a promising novel antibiotic target, as peptides^5,6^, small molecules^7–9^ and antibodies^10,11^ that interact with the complex have bactericidal activity. In *Escherichia coli*, BAM is a five-component complex (BamABCDE, ~203 kDa). The major conserved subunit is BamA, which contains a 16-stranded, transmembrane β-barrel domain embedded in the OM. BamA also contains five polypeptide-transport-associated (POTRA) domains that extend into the periplasmic space^12–15^. The remaining four components, BamB, C, D and E, are accessory lipoproteins associated with the membrane through covalently linked lipid anchors at their N termini^16,17^. Only BamA and BamD are essential for cell viability in *E. coli*, although double deletions of some subunits are lethal, and all five subunits are required for full function in vivo^4^.

The mechanism by which BAM facilitates the folding and insertion of OMPs into the OM remains unresolved, although the dynamic behaviour of BamA appears to play a vital role^15,18–23^. Within the BamA β-barrel, there is a seam or ‘lateral gate’^19^ that has been observed in either a closed (the ‘lateral closed’) conformation^13,14^, or open (‘the lateral open’)^15^ conformation. Stabilisation of the seam by disulfide cross-linking^19^, or extension of one of the β-strands that form it^20^, is lethal in vivo, suggesting that opening of the lateral gate is essential for BAM function, at least for some substrate OMPs, in the context of a living bacterium. In support of the importance of these lateral gate dynamics, the N-terminal strand of the BamA (β1) barrel has been shown to hydrogen-bond to the substrate OMP, possibly via the β-signal in the C-terminal β-strand in both BAM^24,25^ and its mitochondrial homologue, SAM^26^. The POTRA domains of BamA are mobile^23,27,28^ and have been observed in various conformations in structures of BamABCDE^13–15^ and BamACDE^12^, and this mobility is thought to be important for function, as reducing the flexibility between POTRA domains 2 and 3, by introducing a disulfide bond between them, also impairs BAM function in vivo^23^.

In addition to the importance of the dynamic nature of the BAM complex for OMP folding, evidence suggests that BAM may also facilitate OMP folding by disordering or thinning of the lipid bilayer in which it is embedded^29^. This evidence includes (1) a reduced hydrophobic thickness of the BamA β-barrel near its gate^30^, (2) molecular dynamics (MD) simulations that show lipid disorder and membrane thinning near the lateral gate^21,29,30^, (3) the fact that BamA’s activity in vitro depends on lipid type, with activity being higher in bilayers with native lipid headgroups^31,32^, (4) that membrane defects accelerate unassisted OMP folding in vitro^33^ and (5) that BamA’s activity in vitro depends on membrane thickness, with a greater acceleration of BamA-assisted OMP folding in thicker bilayers^29^.

To investigate directly the structural and dynamic properties of BAM in a bilayer environment, we determined the structure of the BAM complex reconstituted into lipid nanodiscs containing *E. coli* polar lipid extract, stabilised by the membrane-scaffold protein (MSP) MSP1D1 using cryo-electron microscopy (cryo-EM). These studies showed that BAM exists as an ensemble of structures in these nanodiscs, in contrast with all previous structural studies of BAM, including cryo-EM studies of BAM in detergent micelles, where a single structure was observed^12–15^. Interestingly, in all the 16 different structures that we obtained BAM in nanodiscs, the complex is found in the ‘lateral open’ conformation, suggesting that this conformation is the most stable state for the BamA β-barrel in BAM in this lipid environment. The structures obtained also portray the POTRA domains in an array of conformations in nanodisc-embedded BAM. The extent of this motion, however, is much smaller than that suggested by comparison of previous X-ray and cryo-EM structures. Using single-molecule FRET (smFRET) experiments, we show that the extent of these POTRA domain motions in solution matches that seen in the ensemble of cryo-EM structures, and that these motions occur on a ms timescale. Remarkably, compared with previous studies of α-helical integral membrane proteins in nanodiscs^34–37^, a dramatic distortion of the MSPs is observed in the BAM-containing nanodiscs. This disruption is particularly pronounced in the region of the MSP proteins adjacent to the lateral gate in BamA. MD simulations of BAM in an MSP1D1 nanodisc mirrored this distortion and showed that it is correlated with BAM-mediated lipid perturbation. Collectively, these results provide the first structure of BAM in a lipid bilayer, along with direct structural evidence that BAM disrupts the membrane bilayer in the vicinity of its lateral gate. This presumably helps to reduce the kinetic barrier to OMP insertion and thereby facilitates OMP folding into the OM.

## Results

**Fig. 1:**
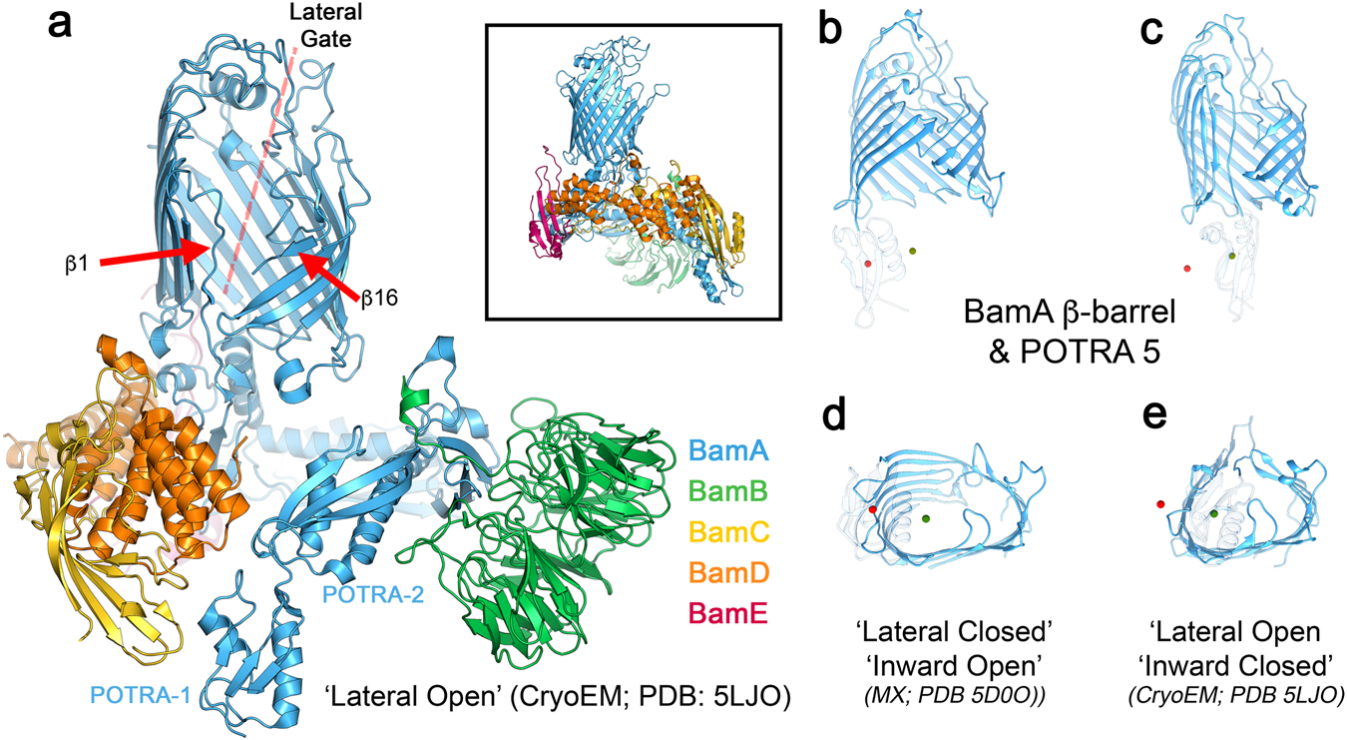
Structure of BAM highlighting its lateral gate. **a** Cryo-EM structure (PDB 5LJO)^15^ of detergent (DDM)-solubilised BAM in the ‘lateral open’ conformation. The β-strands 1 and 16 and the lateral gate are indicated. Inset: the crystal structure (PDB 5D0O)^13^ of BAM in the ‘lateral closed’ conformation. **b** The conformation of the BamA β-barrel and position of POTRA5 in the ‘lateral closed’ X-ray structure (pdb 5D0O)^13^ and **c** ‘lateral open’ cryo-EM structure (pdb 5LJO)^15^. The centres of mass of POTRA5 for both conformations are marked with red (lateral closed) and green (lateral-open) spheres in each panel. **d**, **e** Bottom (periplasmic) view of the **d** lateral closed and **e** lateral-open conformations of BAM showing the different shape of the β-barrel. As in panels **b** and **c**, centres of mass for POTRA5 in both conformations are marked in each panel. POTRA domains 1–4 and the lipoproteins (BamB–E) have been removed in panels (**b**) through (**e**) for clarity.

The seam or ‘gate’ in the BamA β-barrel is formed by the interface between β-strands 1 and 16 (Fig. 1a). In structures of the complete BamABCDE (BAM) complex, this ‘lateral gate’^19^ has been observed in either a closed (the ‘lateral closed’ (Fig. 1b, c)) conformation^13,14^, or open (‘the lateral open’^15^ (Fig. 1d, e)) conformation. The state of the gate is correlated with larger conformational differences throughout BamA. The BamA β-barrel is relatively round (in cross-section) in the lateral-open conformation, but adopts an oval profile in the lateral closed conformation. In addition, POTRA5 is positioned directly below the lumen of BamA’s β-barrel in the lateral-open state, while it repositions to the side of the BamA β-barrel in the lateral closed conformation. This rearrangement opens access to barrel interior from the periplasmic side^13^.

### The BAM complex is functional in lipid nanodiscs

**Fig. 2:**
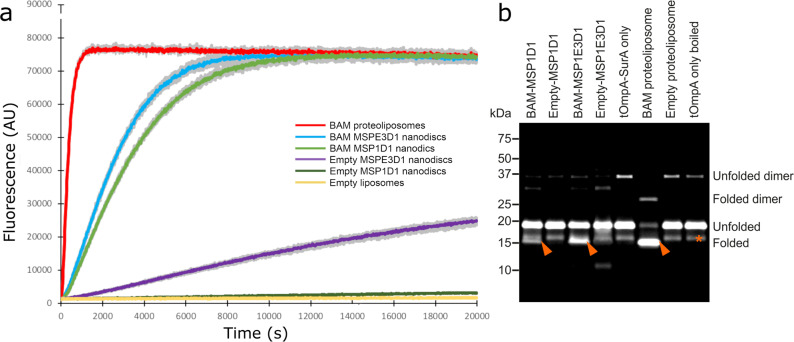
Comparison of the catalytic activity of BAM in proteoliposomes and nanodiscs. **a** Fluorescence detected from cleavage of a fluorescent OmpT substrate in BAM-containing proteoliposomes (red), BAM in MSPE3D1 (blue) or MSP1D1 nanodiscs (green), empty nanodiscs (purple and dark green for MSPE3D1 and MSP1D1, respectively) and empty liposomes (i.e. lacking BAM) (yellow). For each sample, the coloured line represents the mean fluorescence, whilst grey lines represent the minimum and maximum values for three replicates. **b** Gel shift assay measuring BAM-catalysed folding of tOmpA. Folded tOmpA is indicated by red arrows, whilst a contaminating band, present in all samples, is marked with a red asterisk. In this assay, tOmpA is labelled with Alexa Fluor 488, enabling the folding of the substrate to be visualised using fluorescence detection, without interference from protein bands arising from BamABCDE and SurA.

To investigate the structure, function and dynamics of the BAM complex in a lipid bilayer, we reconstituted BAM into lipid nanodiscs containing *E. coli* polar lipid extract (Supplementary Fig. 1) and used enzyme activity with a fluorescent substrate (OmpT, a 10-stranded β-barrel) or fluorescence band-shift assays using sodium dodecyl sulfate-polyacrylamide gel electrophoresis (SDS-PAGE) (tOmpA, an 8-stranded β-barrel) to monitor the formation of folded protein as a measure of BAM activity (see ‘Methods'). BAM is capable of folding both OmpT and tOmpA into MSP1D1 (97-Å diameter) and MSP1E3D1 (129-Å diameter) nanodiscs (Fig. 2a), consistent with previous observations that BAM can fold the 12-stranded autotransporter EspP into MSP1D1 nanodiscs^38^. However, for the two OMPs studied here, BAM-catalysed folding into both nanodiscs was slow and inefficient, compared with the folding of the same substrates into BAM-containing proteoliposomes (Fig. 2). This is exemplified by the folding of tOmpA, which was highly efficient in BAM-containing proteoliposomes (folding yield 84%), but much less efficient in both MSP1E3D1 and MSP1D1 nanodiscs (15% and 39% folding yield, respectively, Fig. 2b), consistent with the activity of BAM being impaired when the lipid environment is physically constrained. Interestingly, OmpT was able to fold spontaneously (albeit with low yield) into empty MSP1E3D1 nanodiscs (i.e. lacking BAM), but not into liposomes formed from *E. coli* polar lipid extract (Fig. 2), possibly reflecting decreased lipid ordering and increased lipid dynamics within these nanodiscs^39^.

### The BAM complex distorts the lipid bilayer

**Fig. 3:**
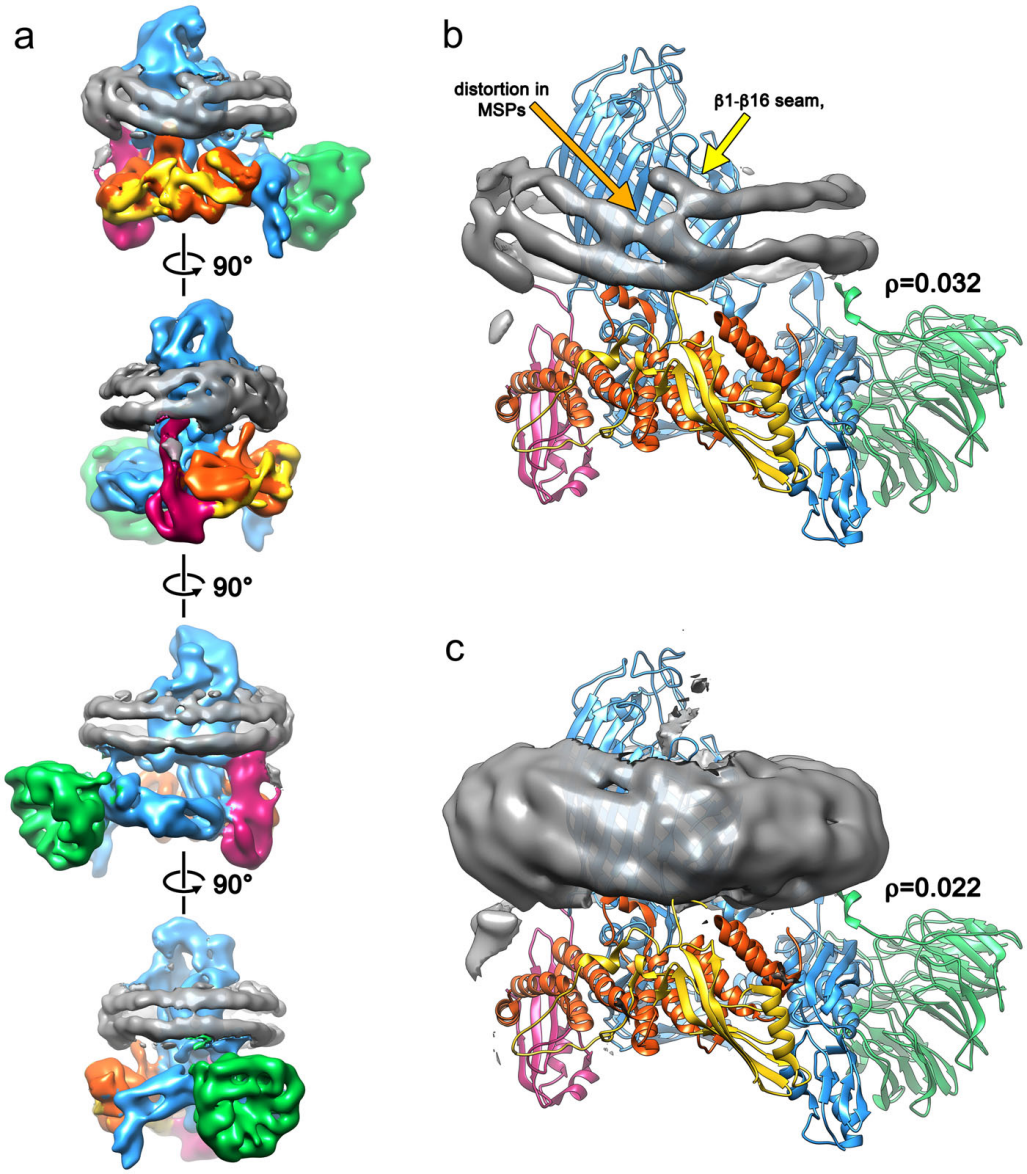
The cryo-EM structure of BAM reveals distortion of the lipid nanodisc MSPs. **a** Cryo-EM map of the consensus structure of the BAM-containing MSP1D1 nanodisc with all five BAM subunits and nanodisc density colour coded as in Fig. 1 and the nanodisc density in grey. Each panel represents a 90° rotation around the axis shown. The cryo-EM density for the membrane-scaffold proteins is shown with the fitted atomic model of the BAM complex at low contour level (*ρ* = 0.032; panel **b**) and high contour level (*ρ* = 0.022; panel **c**) showing distortion of the MSP from the expected planar geometry adjacent to the BamA lateral gate.

We next determined the structure of BAM in MSP1D1 nanodiscs to 6.7-Å resolution (Supplementary Fig. 2) using cryoEM (Fig. 3). This ‘consensus structure’ was calculated from 208,549 individual particle images, contains density for all five-component proteins of BAM and overall is similar to the cryo-EM structure of BAM in detergent micelles (Supplementary Fig. 3)^15^. The two maps have a global cross-correlation coefficient of 0.95 when the micelle/nanodisc density is subtracted (see Methods). BamA, B, D and E are well resolved, while only the N-terminal ‘lasso’^40^ region of BamC is represented by strong density. The C-terminal, globular domains of BamC are weak and poorly resolved, suggesting that these domains are mobile in the reconstituted complex, consistent with previous X-ray crystallography and cryo-EM structures^12–15^. The gate of the BamA β-barrel is in a ‘lateral-open’ conformation, and the POTRA domains are in a similar conformation in the cryo-EM structure of BAM in detergent micelles^15^. The N-terminal regions of BamB and BamE make contact with the lipid bilayer, consistent with the location of their lipid anchors^15^. Additional interactions with the membrane are observed by a loop on POTRA3 composed of BamA_200–213_, as predicted from simulations of BamA in a native membrane^41^, and a 3_10_ helix composed of BamD_123–129_ (Supplementary Fig. 4), and consistent with predictions based on our previous cryo-EM structure of detergent-solubilized BAM^15^.

The consensus structure of BAM has an additional, fascinating feature in that the MSPs that form the nanodisc are resolved as two contiguous belts of density around the periphery, each of which can accommodate the single α-helix of the MSP (Fig. 3). This density is distorted adjacent to the lateral gate in the BamA barrel (Fig. 3), and close to BamD, the only BAM lipoprotein that is essential for viability and is widely conserved across proteobacteria^42^. Indeed, the membrane-interacting 3_10_ helix enters the membrane precisely in the distorted region of the nanodisc. Ordered lipid molecules are not observed, and no part of the BamA β-barrel appears to be physically in contact with the MSPs. The only region where density appears that could suggest an interaction between BAM and the MSP is in the loop comprising residues 184–214 of BamA and the N terminus of BamE (Supplementary Fig. 5). We were not able to model the N-terminal lipid anchors of BamB–D, but again the lack of strong density or these regions suggests that they are not highly ordered. Overall, this suggests that distortion of the nanodisc MSP is mediated by lipid molecules, such that destabilization of the bilayer by the BAM complex allows the MSP proteins to sample more conformational space in the region adjacent to the lateral gate, leading to the EM structure presented here with MSP distortion and the lateral gate co-located. Most cryo-EM reconstructions of protein-containing nanodiscs do not show distortions (Supplementary Table 1), although recent cryo-EM structures of the lipid scramblase nhTMEM16 in lipid nanodiscs also reported deviation from planarity in the MSPs. This was attributed to distortion of the membrane by the inserted protein^43,44^, and it was hypothesized that the resulting deformation was necessary for the protein’s function.

It has yet to be seen whether this MSP distortion is specific to BAM, or might occur with other OMPs, but a recent structure of the sorting and assembly machinery of the mitochondria (SAM) in a larger MSP1E3D1 nanodisc does not show similar distortion^45^. SAM contains the BamA homologue Sam50, but has different accessory proteins. The lateral gate of the Sam50 β-barrel is observed in multiple conformations resembling both the lateral open and lateral closed BamA gate conformations, but SAM is locked in a lateral closed conformation by the other metrics used here to determine the conformation of BAM. The diameter of the β-barrel stays constant between the different conformations, and SAM’s single, non-essential^46^, POTRA domain remains in a position akin to that in the lateral closed BAM, leaving the lumen of the β-barrel unoccluded and accessible from the cytoplasmic side of the complex^45^.

### MD simulations show membrane distortion in BAM nanodiscs

**Fig. 4:**
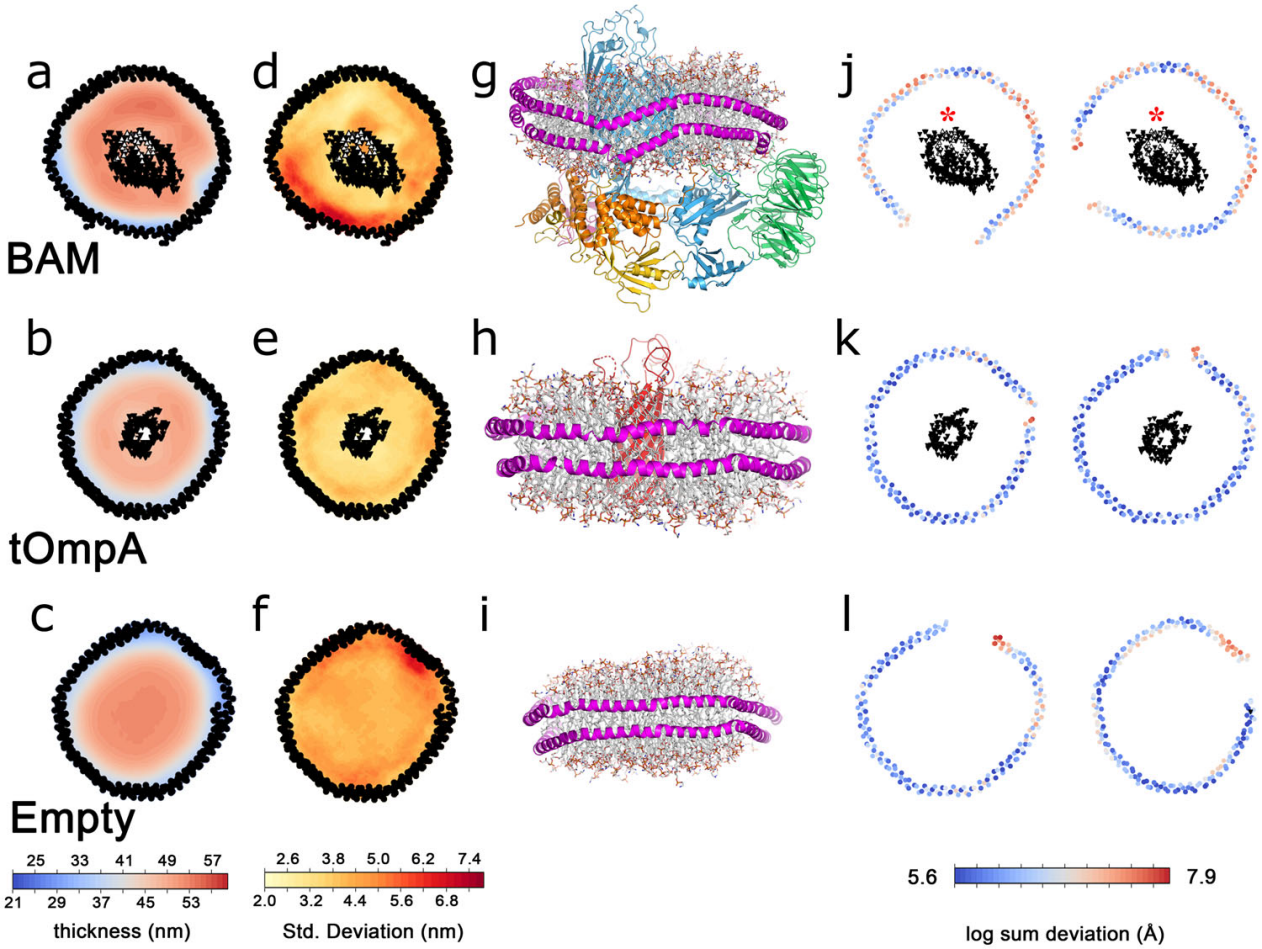
Molecular dynamics simulations of protein-containing and empty lipid nanodiscs. Mean thickness of lipid bilayers over the course of a 1-µs simulation for BAM-containing (**a**), tOmpA-containing (**b**) or empty (**c**) MSP1D1 nanodiscs. The standard deviation of bilayer thickness over the 1-μs simulation for BAM-containing (**d**), tOmpA-containing (**e**) or empty (**f**) MSP1D1 nanodiscs. Residues in the lateral gate are indicated in white. The 3D structures of the final frame of each simulation, coloured as in Fig. 1, with the MSP in magenta for BAM-containing (**g**), tOmpA-containing (**h**) or empty (**i**) MSP1D1 nanodiscs. Deviation from planarity for the upper (left) and lower (right) MSP in BAM-containing (**j**), tOmpA-containing (**k**) or empty (**l**) MSP1D1 nanodiscs. In **a**, **b**, **d**, **e**, **j** and **k** the mean positions of the α-carbons of the MSPs and β-barrels of the inserted proteins are represented by black dots. Strands β1 and β16, which make up the lateral gate of BamA, are indicated with a red asterisk in (**j**).

To further investigate the role of BAM in bilayer distortion, we performed a 1-µs all-atom MD simulation of the intact BAM complex in an MSP1D1 nanodisc, using a lipid mixture that mimics *E. coli* polar lipid extract (Methods and Supplementary Table 2). Similar MSP distortions to those seen in the cryo-EM structure are observed in the simulation, with distortions occurring within ~40 ns of starting the simulation (Fig. 4a, d, g, j (top row)). Crucially, no such distortions were observed in control simulations containing the 8-stranded β-barrel protein tOmpA (the transmembrane domain of OmpA)^47^ (Fig. 4b, e, h, k (middle row)) or in an empty nanodisc (i.e. containing lipid, but no protein) (Fig. 4c, f, i, l (lower row)), each with identical lipid mixes. To characterise the behaviour of the lipids in these simulations, we measured membrane thickness as a function of distance from the lipid headgroups to a plane equidistant from the two leaflets and parallel to the orientation of the nanodisc over the course of the simulation (Methods). Consistent with previous results, for the empty nanodisc, a centrosymmetric increase in membrane thickness is observed with distance from the MSPs^48–50^ (Fig. 4c). In the simulation of a tOmpA-containing nanodisc, there is a similar centrosymmetric increase in membrane thickness with distance from the MSP (Fig. 4b). Strikingly different behaviour is seen in the simulation of a nanodisc that contains the BAM complex, where the distribution of membrane thickness is complex, variable and highly asymmetric (Fig. 4a). On the side of the BamA β-barrel adjacent to the β1–β16 seam, the membrane is the thinnest nearest the MSPs and increases towards the BamA barrel, whilst near the β1–β16 seam, thinning is observed around the lateral gate, consistent with previous simulations of the BamA β-barrel (i.e. without BamBCDE) in a bilayer^21,29,30^. The membrane is the thickest on the side of the BamA β-barrel furthest from the lateral gate, where the bilayer is on average up to 10 Å thicker than in the area close to the MSP nearest to the gate.

The MSP proteins are even more distorted in the BAM-nanodisc simulation than in the consensus EM structure, but follow a similar pattern, with the nanodisc distorted out of the plane near the lateral gate (Supplementary Fig. 6). Given that no direct contacts are observed between BAM and the MSP in the cryo-EM structure, the results suggest that distortions result from disruptions to the lipid bilayer induced by BAM.

### BAM in a nanodisc is in an ensemble of conformations

**Fig. 5:**
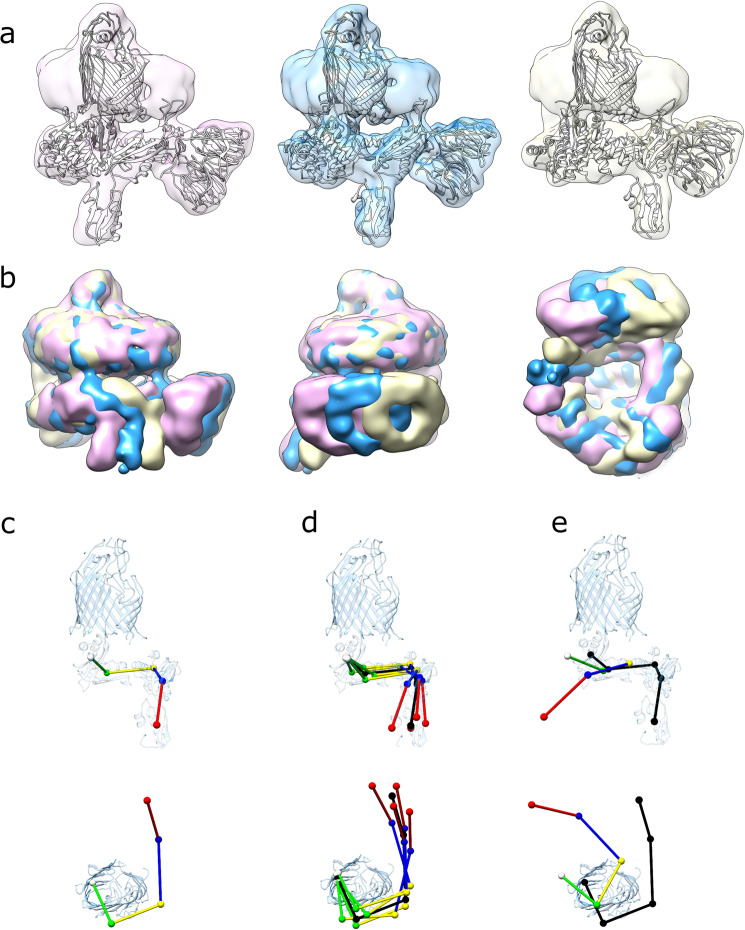
Cryo-EM structures generated by separation along eigenvectors 0 and 1. **a** EM maps of (left to right) conformations 0–1, 0–5 and 0–9, with fitted models. Each reconstruction is aligned on the β-barrel of BamA illustrating the difference in orientation of the bottom half of the complex. **b** EM maps of conformations 0–1 (pink), 0–5 (gold) and 0–9 (blue) aligned on the β-barrel of BamA and overlaid. **c** Positions of centres of mass for POTRA domains 1 through 5 (red, blue, yellow, green and white, respectively) in the BAM-nanodisc consensus structure. **d** Positions of the POTRA domains in the four extremes of the ensemble (0–1, 0–9, 1–2 and 1–8, respectively, coloured) compared with the consensus structure (black). **e** Comparison of the positions of the POTRA domains in the ‘lateral closed’ crystal structure (5D0O^13^, coloured) with the BAM-nanodisc consensus structure (black).

The consensus structure of BAM in MSP1D1 nanodiscs at 6.7-Å resolution contains ~208 k particle images, far more than the ~96 k images used to calculate the 4.9-Å resolution structure of BAM in dodecyl maltoside (DDM) micelles^15^. To investigate why higher resolution could not be achieved despite the large dataset employed, an extensive set of focused refinements was performed using multibody refinement in RELION^51^. This approach is generally used to consider the movement of independent rigid bodies relative to each other in large protein complexes. Principal component analysis of the multibody refinement of BAM as two distinct halves (the membrane-embedded and periplasmic regions, Supplementary Fig. 7a) identified 6 component motions, accounting for 85% of the variability in the dataset (Supplementary Table 3, Supplementary Movies 1–6). However, such refinements did not improve the resolution of the overall reconstruction. The eigenvalues for these six main principal components form wide, monomodal distributions around the consensus structure, suggesting continuous variability rather than discrete states^51^ (Supplementary Fig. 13). Ultimately, we arbitrarily subdivided the data into 16 classes based on the eigenvalues of the first two components (components 0 and 1) (Fig. 5), and an EM map was created from each of the resulting subgroups (Supplementary Table 4), generating low-resolution snapshots of conformations present in the data. Component motion 0 (~20% of the variability), is a rotation of the bottom half of the complex (including the POTRA domains and BamB, C, D and E) around an axis approximately perpendicular to the plane of the membrane (Supplementary Fig. 7c). Component motion 1 (~17% of the variability) is a tilting of the bottom half of the complex around an axis approximately parallel to the plane of the membrane (Supplementary Fig. 7d). In all classes, the eigenvalues for components that were not selected on were distributed around means consistent with the dataset as a whole, suggesting that the eigenvalues of components 0 and 1 are independent of each other, and of all other principal components (Supplementary Fig. 8). If BAM populated discrete conformations, we would expect to find multimodal distributions. Collectively, therefore, these data demonstrate that BAM in a nanodisc is a continuously variable structure.

In total, 17 reconstructions of the BAM structure were generated: the consensus map at 6.7 Å, and 16 reconstructions (8.4–10.8-Å resolution) that represent classes generated by separating the data on eigenvalues of components 0 or 1 (Supplementary Table 4, Supplementary Fig. 9). Atomic models were flexibly fitted into all 16 maps, giving snapshots of the conformations in the larger ensemble. There is no evidence of any ‘lateral closed’ barrel conformation (although more subtle conformational differences cannot be ruled out at this resolution). Although the lateral gate is difficult to visualise at these resolutions, the overall shape of the barrel and the position of POTRA5 (directly below the lumen of the BamA β-barrel) is consistent with X-ray^12,13^ and EM^15^ structures of the lateral closed conformation, in all models generated (Supplementary Fig. 10). Similarly, the accessory lipoproteins BamB–E are essentially invariant at the resolution of the 16 different structures. The most dramatic differences are in the positions of the five periplasmic POTRA (P) domains. In all structures (here and previously published^12–15^), these form a ‘corkscrew’ projecting into the periplasm. In the 16 structures determined here, the position of P5, which is closest to the base of the BamA barrel, is altered by small translations/rotations (Supplementary Fig. 11) that subtly alter the position of the ‘corkscrew’ relative to the BamA β-barrel. There are also changes in the angular relationships between individual POTRA domains, although the relative motions of P4–P5 (maximum 2.4 Å), P3–P4 (maximum 2.5 Å) and P2–P3 (maximum 2.7 Å) are small compared with the movements of P5 relative to the BamA β-barrel (maximum 5.4 Å) (Supplementary Fig. 12). Collectively, these small conformational shifts lead to the propagation of larger conformational changes along the length of the POTRA chain. P1 is relatively unconstrained and is very mobile, with maximum overall displacements of ~25 Å in its centre of mass within the ensemble, although the maximum movement relative to P2 is 6.4 Å (Fig. 5, Supplementary Fig. 12, and Supplementary Movies 1 and 2).

### The ensemble of EM structures is consistent with smFRET

**Fig. 6:**
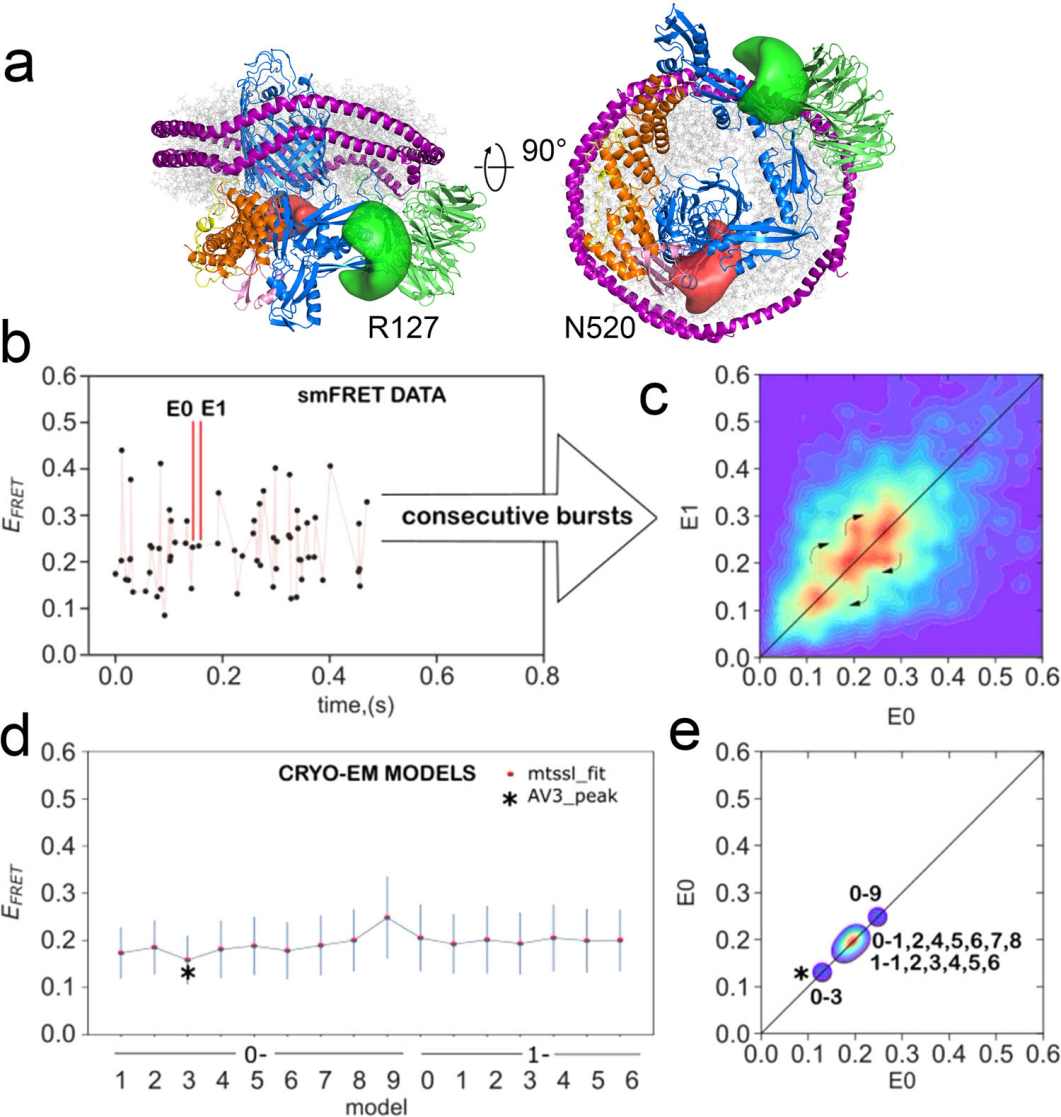
Comparison of experimentally measured and predicted smFRET values of BAM in MSP1D1 nanodiscs. **a** Structure of a dye-labelled double-cysteine variant of BAM (R127C/N520C) predicted from the previous MD simulations. Green and red regions represent possible space filled by Alexa Fluor 488 and Dylight 594 maleimide, respectively. **b** Representative time trace showing *E*_FRET_ values for single molecules. **c** Experimental 2D transfer-efficiency contour plot (RASP analysis, see Methods) showing the measured *E*_FRET_ between the dyes for pairs of consecutive bursts E0 and E1 with d*t* < 30 ms. Density on the diagonal line indicates persistent conformational states, while off-diagonal density indicates transitions between those states that occur on a 1–30-ms timescale (curved arrows). **d** Predicted *E*_FRET_ for each of the 16 cryo-EM-derived model structures; each point represents the maximum of the predicted distribution as shown for selected structures in Supplementary Fig. 13a. **e** Projections of the predicted *E*_FRET_ into a 2D plot for direct comparison with the experimental data (panel **b**) showing that the static pattern of density along the diagonal could be recapitulated by the 16 cryo-EM-derived model structures. An alternative modelling approach afforded a better match for the data in the case of one structure (0–3 black star).

We next sought to assess whether the range of conformations observed using cryo-EM accurately captures the conformational ensemble found in solution, using smFRET. We, therefore, prepared a double-cysteine variant of BAM (R127C and N520C) in which the two natural cysteine residues (C_690_ and C_700_) were replaced with serines, and two new cysteines were introduced into BamA, one in POTRA2 (R127C) and the other in a loop between β-strands 6 and 7 at the bottom of the β-barrel (opposite the lateral gate (N520C)). These cysteines were labelled stochastically with Alexa 488 and DyLight 594 (Fig. 6a), allowing POTRA domain dynamics relative to the BamA β-barrel to be measured in real time in freely diffusing MSP1D1 nanodiscs using smFRET confocal microscopy with 100-μs temporal resolution. As individual molecules diffuse through a femtolitre laser beam confocal volume, analysis of the emitted fluorescence in the donor (D) and acceptor (A) channels yields *E*_FRET_ values proportional to interdye distance:

$$E_{\rm{FRET}} = \frac{1}{{1 + \left( {\frac{R}{{R_0}}} \right)^6}} = 1 - \frac{{F_{\rm{DA}}}}{{F_D}}.$$

The distribution of observed *E*_FRET_ values is shown in Fig. S13a and the distribution of *E*_FRET_ predicted for each of the 16 cryo-EM-derived model structures is overlaid. In order to make a clear visual comparison between the E_FRET_ observed experimentally and predicted for the conformations recovered from the cryo-EM, we employed recurrence analysis of single particles.

At low picomolar concentration, consecutive fluorescent signal bursts occurring within a few tens of milliseconds are likely (*p* > 0.95) to correspond to multiple passes or recurrence^52^ of a single molecule through the small volume (Supplementary Fig. 13 and Fig. 6b, c). *E*_FRET_ values for each pair of consecutive bursts ‘E0’ and ‘E1’ that were separated by less than 30 ms were plotted, yielding a 2D transfer-efficiency contour plot (Fig. 6c). Regions of density on the diagonal reflect persistent, abundant conformations in the ensemble, while off-diagonal cross-peaks reflect transitions between these conformations. The range of interburst times observed for bursts attributed to recurrence defines an approximate timescale for the conformational change from 1 to 30 ms (Supplementary Fig. 13f). Recurrence analysis thus provides a simple, model-free way to resolve conformational states and transitions between them^52^. The 16 structures in the cryo-EM ensemble were used to simulate the expected *E*_FRET_ values of these conformations (Fig. 6d, e) (Methods) using well-established coarse-grain simulation approaches^53,54^. A 2D contour plot of the predicted *E*_FRET_ values calculated from the ensemble of observed cryo-EM structures (Fig. 6e) closely resembles the observed pattern of on-diagonal density (states) observed experimentally. The continuous motion of the POTRA domains relative to the barrel causes the average position of each dye to move relative to each other along curvilinear paths. In contrast, the *E*_FRET_ arises from the linear interdye distance as the dyes move along their respective curvilinear paths. In addition, steric factors that influence the space available to the dyes will be different for different conformations. Thus, although the majority of the cryo-EM-derived structures have very similar *E*_FRET_ ~0.2, two of the cryo-EM-derived models show a notably higher (0–9 EFRET~0.25) and notably lower (0–3 *E*_FRET_ ~0.13), which could explain the three *E*_FRET_ states revealed by the RASP analysis.

BAM reconstituted into MSP1D1 nanodiscs; therefore, samples conform in a solution that is consistent with those observed in the cryo-EM ensemble, with transitions between conformations occurring on a few millisecond timescales.

## Discussion

Much progress has been made in understanding OMP biogenesis^1,2,4,24–26^, with important contributions from in vivo and in vitro studies on the chaperones involved^55^, and the architecture of BAM^12–15^. In particular, structures of the BAM complex in detergent in the ‘lateral open’ and ‘lateral closed’ conformations (Fig. 1), suggest that interconversion between these states may be required to catalyse OMP folding into the OM^12–15^. Interactions between β1 and substrate^24^, and between substrate and BamA’s β-barrel,^25^ suggest that the ‘lateral closed’ state may be a substrate acceptor state. However, locking BAM into a ‘lateral closed’ conformation, by extending β16^20^ or by binding an antibiotic^9^, is bactericidal. This suggests roles for both conformations, and their interconversion, in the BAM functional cycle. However, the molecular details of how BAM catalyses OMP folding into the OM remain poorly understood^1,2,4^.

The 17 structures of BAM in a nanodisc presented here represent snapshots of a complex exhibiting continuous variability. In all of these structures, the BamA is in the ‘lateral open’ conformation, and we find no evidence for gate closing in nanodiscs using smFRET, cryo-EM or in MD simulations. The variability arises from the movement of the POTRA domains relative to the barrel, with minor contributions arising from motions of the POTRA domains relative to each other. Structural data on pairs of isolated POTRA domains^28,56,57^, POTRA domains in BamA alone^27,41^ and in various structures of the intact BAM complex in detergent^12–15^ suggested that this region was flexible. Here, we provide direct evidence for this flexibility in a lipid bilayer. POTRA5 moves most, with the overall twist of P5–P4–P3 being largely invariant (Supplementary Fig. 12)^23,27^. Interestingly, the extreme differences in orientation of the POTRA domains observed in BamACDE^13^ are not observed in the ensemble of structures in nanodiscs, possibly because BamB is required for such changes, or because such changes are stabilized by crystal-packing interactions (Supplementary Fig. 14).

The β-barrel of BamA is in a ‘lateral-open’ conformation in all the structures presented here, suggesting that the ‘lateral open’ conformation is a low-energy state, at least in *E. coli* polar lipid nanodiscs and DDM micelles^15^. Thus, apo-BAM might exist in the OM as a continuously variable ensemble weighted towards the ‘lateral open’ conformation (although this may not be the case in the asymmetric OM). Importantly, the motions captured by cryo-EM and smFRET of BAM in nanodiscs are small compared with those needed to achieve the ‘lateral closed’ structure^13,14^ (Fig. 5c–e). We found no evidence for a ‘lateral closed’ conformation in our data. In addition, although variability in the conformation of the lateral gate and POTRAs of BamA was observed in the MD simulations of BAM in a nanodisc, the range of motion on the timescale of the simulation was consistent with the lateral-open state (Supplementary Fig. 15). Recent experiments with stalled substrate complexes suggest that BamA’s β1 strand faces inwards allowing interactions with the β-signal-containing C-terminal β-strand of an incoming substrate OMP^24,25^, and requiring a conformation much closer to the ‘lateral closed’ state^13,14^. This suggests that a conformational change in the gate, coupled with the movement of POTRA5, may occur upon the initial encounter between a substrate OMP (or OMP/chaperone complex) with BAM, or that a low population closed state exists within the cryo-EM dataset. The flexibility of BAM reported here suggests that such a change could occur by shifting the equilibrium position of the complex, rationalizing how OMP delivery to BAM and folding/release into the OM can occur without an external energy source such as the ATP hydrolysis used by other protein-folding machines^58^. Indeed, dynamics at the ‘lateral gate’ are clearly possible, as disulfide-trapping experiments show that β1 and β16 of BamA can undergo register sliding of >14 Å, both in vivo and in vitro^20,22^. In the MD simulations, the mobile part of the lateral gate (composed of β1, loop1 and β2) also adopted several novel conformations that appear to have a greater amount of contact with β16. Although overall these structures are unambiguously ‘lateral open’ based on the position of POTRA5 and the shape of the BamA β-barrel (Supplementary Fig. 15), they raise the possibility of additional variations on the lateral-open conformation. Recent cross-linking data also suggest that the inside surfaces of the BamA β-barrel may catalyze OMP folding^25^. However, the roles of lipid dynamics, bilayer asymmetry and the order of different states in the BAM functional cycle remain to be resolved.

Lipid nanodiscs allow observation of functional membrane proteins in a bilayer, the effects of membrane on protein^35,59^ and vice versa^43^. However, they are far from perfect analogues of biological membranes. The folding activity of the BAM complex in MSP1D1 nanodiscs is substantially lower than when reconstituted into proteoliposomes (Fig. 2), a phenomenon also seen with other integral membrane proteins^39^. BAM in the larger MSP1E3D1 nanodiscs was more active than in MSP1D1 nanodiscs, but was still less active than BAM in proteoliposomes (Fig. 2). ssNMR^39^, SANS^60^ and MD simulations^48–50,61,62^, all suggest that lipids are more closely packed in MSP nanodiscs than in liposomes, and this may affect the function of embedded-membrane proteins, especially those like BAM, which induce disorder in bilayers to deliver their function. The liposomes used here were 100–200 nm in diameter, giving them more curvature than flat nanodiscs, which could also impact BAM function. Maximally efficient OMP folding may also require BAM oligomers as ‘islands’^63^ or ‘precincts’^64^, or an excess of lipid into which the OMP can fold (a lipid:protein ratio of ~1000:1 (mol/mol) is used in folding assays with BAM-containing liposomes, compared with ~60:1 (mol/mol) for BAM in MSP1D1 nanodiscs. Indeed, we saw no evidence for multiple BAMs incorporated into the nanodiscs, as expected given their size (9.7-nm diameter) compared with the diameter of BAM (the periplasmic region of the complex being ~11 nm at its widest point). This would also limit the number of substrate proteins that can be folded into a given nanodisc, effectively meaning the functional assays are a single-turnover format compared with that in BAM proteoliposomes. Finally, it is worth noting that the *E. coli* polar lipid mix does not mimic the OM, which has lipopolysaccharide (LPS) in its outer leaflet. We did not directly test for LPS in our BAM preparations, but saw no evidence for structured lipids in the EM maps. How this asymmetry that alters the conformation and dynamics of the BamA β-barrel remains unknown.

Several models have been proposed to explain how BAM mediates OMP folding^2,4,24,25^. These include the ‘BamA-assisted’ model, where BAM-catalysed OMP is similar to unassisted folding, albeit into a BamA-destabilised membrane;, a ‘budding’ model, where a BamA-substrate OMP hybrid barrel structure is formed^1,4,19^, a ‘barrel-elongation’ model, proposing that substrate interaction with BamA β1 nucleates OMP β-sheet structure within the BAM periplasmic ring^4^, a ‘swing’ model, where conformational changes in BAM pull a partially folded OMP into the bilayer^24^ and an ‘interior surface’ model where the inside of the BamA barrel catalyzes OMP β-sheet formation^25^. Recent in vivo data in mitochondria^26^ and in bacteria^24^ using stalled substrate complexes show that β1 strand of BamA (and its mitochondrial homologue Sam50) interacts with the C-terminal strand of nascent OMPs during folding^24–26^, consistent with all of these models. Crucially, however, all models generally include BAM-induced membrane destabilization to reduce the kinetic energy barrier for insertion of a folding OMP into the bilayer^30,31^.

The 6.7-Å resolution consensus structure and MD studies reported here for BAM in MSP1D1 nanodiscs reveal deformation of the nanodisc MSP uniquely caused by BAM, and thus give direct insights into BAM-induced membrane destabilization. This distortion is the greatest adjacent to the BAM lateral gate in both the consensus EM structure and in MD simulations (Figs. 3 and 4). Furthermore, in MD simulations, the bilayer is profoundly disturbed relative to that seen in nanodiscs lacking protein, or in nanodiscs containing tOmpA. BAM induces local defects in the membrane^30^ by the irregular shape of its β-barrel, and has been proposed to reduce the kinetic barrier to OMP folding imposed by specific lipid headgroups^31^. OMP folding is also sensitive to the lipid environment, with increased folding rates observed in membranes expected to have more defects, such as those with curvature^65^, thinning^65^ and at their phase-transition temperature^33^. A similar deformation was observed previously in nanodiscs containing the scramblase TMEM16, which induces membrane distortions to assist in guiding lipids into its active site^43^. For BAM, such lipid distortion is proposed to be required for its function^24,30,31,33^. The BAM-nanodisc structures described here thus provide direct evidence that BAM acts as a membrane ‘disruptase’, which might help all OMPs to fold into the OM, regardless of a need to utilize the BAM lateral gate to further catalyze folding. Whether some OMPs rely solely on this lipid disruption will be an important question for future studies. Irrespective of the answer, the results presented here highlight the importance of BAM’s lipid ‘disruptase’ activity for OMP folding and presumably for the biogenesis of the *E. coli* OM.

## Methods

### Preparation of BAM in lipid nanodiscs

Preparation of detergent-solubilized BAM complex and cysteine mutants: The intact BAM complex (BamABCDE) or BamA(R127C/N520C/C690S/C700S)BCDE was expressed and purified as described previously adapted from Roman-Hernandez et al.^66^. *E. coli* BL21(DE3) was transformed with plasmid pJH114 containing BamA–E with a C-terminal His_6_ tag on BamE. Cells were grown in 2× TY broth (37 °C, 200 rpm) to an OD_600_ of ~0.6, and protein expression induced with 0.4 mM IPTG. Following 1.5 h of expression, cells were harvested by centrifugation in a Beckman JLA-8.1000 rotor (4000 rpm, 15 min, 4 °C), the pellet resuspended in 20 mM Tris-HCl, pH 8, lysed with a cell disruptor (Constant Cell Disruption Systems, UK), then centrifuged (6000*g*, 10 min, 4 °C) to remove cell debris. Cell membranes were isolated by ultracentrifugation in a 50.2Ti rotor (45,000 rpm, 30 min, 4 °C). The pelleted membranes were then incubated at 4 °C for 2 h with 10 ml/L cold 50 mM Tris-HCl, pH 8.0, 150 mM NaCl (TBS), 1% (w/v) DDM and the ultracentrifugation repeated to remove insoluble material. The BAM complex was isolated by Ni-affinity chromatography using Ni-NTA agarose beads and purified by size-exclusion chromatography (SEC) on a Superdex 200, 10/300 GL column in TBS, pH 8.0 with 0.05% (w/v) DDM as described previously^15^.

Preparation of MSP1D1 and MSP1E3D1: MSP1D1^67^ and MSP1E3D1^68^ were prepared as previously described^69^. *E. coli* BL21 (DE3) cells were transformed with the pET-28a(+) vector containing the His-tagged MSP insert. Single colonies were used to inoculate 100 ml of starter cultures (LB with 50 μg/ml kanamycin). After overnight growth (37 °C, 200 rpm) starter cultures were diluted into 1 L of LB (1 in 100 dilutions) containing kanamycin (50 μg/ml). Cells were grown (37 °C, 200 rpm) to an OD_600_ of ~0.6, and protein expression induced with 1 mM IPTG. After 3 h, cells were harvested by centrifugation, resuspended in 20 mM sodium phosphate buffer, pH 7.4, containing 1 mM PMSF (15 ml/L of culture), and deoxyribonuclease I (5 mg) and Triton X-100 (1% (v/v)) were added. The cells were homogenized and lysed by sonication (6 × 1-min bursts with 1 min of cooling on ice between each burst). The lysate was centrifuged (30,000*g*, 10 min, 4 °C) and the supernatant applied to a HisTrap FF column (5 ml, GE Healthcare) equilibrated with 20 mM sodium phosphate buffer, pH 7.4. The column was washed with ~100 ml each of Buffer 1 (40 mM Tris-HCl, 0.3 M NaCl and 1% (v/v) Triton X-100, pH 8.0), Buffer 2 (40 mM Tris-HCl, 0.3 M NaCl, 50 mM sodium cholate and 20 mM imidazole, pH 8.0) and Buffer 3 (40 mM Tris-HCl, 0.3 M NaCl and 50 mM imidazole, pH 8.0) before elution of MSP1D1 or MSP1E3D1 with 25 ml of 40 mM Tris-HCl, 0.3 M NaCl and 0.4 M imidazole. For nanodiscs used in BAM activity assays, the His tag was cleaved by addition of TEV protease (1 mg of TEV per 18 mg of MSP) in TBS, pH 8.0, 14.3 mM β-ME for 24 h at 4 °C. The cleaved His tag and TEV protease were removed on a 5-ml HisTrap FF column before overnight dialysis against TBS, pH 8.0 at 4 °C. The purified MSP1D1 or MSP1E3D1 was dialyzed against 20 mM Tris-HCl, 0.1 M NaCl and 0.5 mM EDTA, pH 7.4, overnight at 4 °C, and then concentrated, filtered and snap-frozen in liquid N_2_ for storage at −80 °C.

Assembly of BAM-containing nanodiscs: *E. coli* polar lipid extract (Avanti Polar Lipids, Alabaster, AL, USA) was dissolved at 25 mM in 100 mM sodium cholate in TBS at pH 7.0. For BAM nanodiscs, DDM-solubilized BAM, MSP and solubilized *E. coli* polar lipid extract were combined in 1:3:60 and 1:3:180 (mol/mol/mol) ratios for MSP1D1 and MSP1E3D1, respectively. For empty nanodiscs, the ratios of MSP to *E. coli* polar lipid extract were 1:35 and 1:75 (mol/mol) for MSP1D1 and MSP1E3D1, respectively. The final sodium cholate concentration in the reconstitution mixture was 14 mM. To remove the detergent and promote nanodisc formation, the mixture was incubated with BioBeads (SM-2 (BioRad)) for a total of 24 h at 4 °C, with the BioBeads being replaced a total of 4 times. For BAM in His-tagged cleaved MSP1D1 or MSP1E3D1 used in activity assays, BAM-containing nanodiscs were immobilized on Ni-NTA agarose to remove any empty nanodiscs. Following elution in TBS, pH 8.0, 500 mM imidazole, 5% (v/v) glycerol BAM-containing nanodiscs were dialyzed against TBS, pH 8.0, overnight at 4 °C in 500 µL of Slide-A-Lyzer^TM^ dialysis cartridges MWCO 20 k (Thermo Scientific). The presence of all five BAM subunits in the nanodiscs was confirmed by SDS-PAGE (Supplementary Fig. 1).

### BAM activity assays

Preparation of liposomes: DDM-solubilized BAM was reconstituted into proteoliposomes as described previously^15^, using a procedure modified from Thoma et al.^70^. DDM-solubilized BAM and *E. coli* polar lipid films solubilized in TBS with 0.05% (w/v) DDM were mixed at a 2:1 (w/w) ratio of lipid to protein and dialyzed against detergent-free buffer (20 mM Tris-HCl, pH 8.0, 150 mM KCl and 0.01% (w/v) sodium azide (dialysis buffer)) at 21 °C for 2 days, with a total of four buffer changes. Proteoliposomes were pelleted by ultracentrifugation (100,000*g*, 30 min, 4 °C) and resuspended in TBS, pH 8. The ultracentrifugation step was repeated before the final resuspension in TBS, pH 8, and quantification using a BCA assay (Thermo Scientific).

Expression and purification of SurA: SurA with an N-terminal 6× His tag and a TEV cleavage site was expressed and purified using a protocol adapted from Burmann et al.^71^. SurA was expressed in *E. coli* BL21(DE3) cells, which were lysed with a cell disruptor, and debris removed by centrifugation. SurA in the clarified lysate was immobilized on a 5-mL HisTrap FF column (GE Healthcare), denatured and washed on-column with 25 mM Tris-HCl, 6 M guanidine-HCl, pH 7.2, refolded on-column by washing in 25 mM Tris-HCl, pH 7.2, 150 mM NaCl and 20 mM imidazole and eluted in 25 mM Tris-HCl, pH 7.2, 150 mM NaCl and 500 mM imidazole. Eluted SurA was dialyzed against TBS, pH 8.0, overnight at 4 °C. The His tag was cleaved by addition of 14.3 mM 2-mercaptoethanol and His-tagged TEV protease, produced as previously described^29^. Following incubation for ~18 h at 4 °C, the cleaved His tag and TEV protease were removed on a 5-mL HisTrap FF column. Purified SurA was dialyzed against 5 L of TBS, pH 8.0, concentrated to ~200 µM using Vivaspin 20 MWCO 10-kDa concentrators (Sartorius, UK), aliquoted, snap-frozen in liquid nitrogen and stored at −80 °C.

Expression and purification of OmpT and Cys-tOmpA: OmpT and tOmpA were expressed as inclusion bodies in *E. coli* BL21(DE3) cells, using a procedure modified from McMorran et al.^55^ An N-terminal Cys residue was introduced into tOmpA using Q5 site-directed mutagenesis (NEB). Cells were isolated by centrifugation, lysed by sonication and the insoluble material isolated by centrifugation. The insoluble fraction was resuspended in 50 mM Tris-HCl, pH 8.0, 2% (v/v) Triton X-100, and incubated for 1 h at room temperature (~23 °C) with gentle agitation. The insoluble inclusion bodies were pelleted and washed twice by resuspending in 50 mM Tris-HCl, pH 8.0, and incubating for 1 h at room temperature with gentle agitation. The inclusion bodies were then solubilized in 25 mM Tris-HCl, 6 M guanidine-HCl, pH 8.0 and centrifuged (20,000*g*, 20 min, 4 °C). The supernatant was filtered with a 0.2-µm polyvinyl difluoride (PVDF) syringe filter (Sartorius, UK) and purified by SEC using a Superdex 75 HiLoad 26/60 column (GE Healthcare) equilibrated with 25 mM Tris-HCl, 6 M guanidine-HCl, pH 8.0. The purified protein was concentrated to >200 µM using Vivaspin 20 (5-kDa MWCO) concentrators (Sartorius, UK), snap-frozen in liquid nitrogen and stored at −80 °C. For folding experiments, OmpT and tOmpA were buffer-exchanged into TBS, pH 8.0, 8 M urea using Zeba^™^ Spin Desalting Columns, 7 k MWCO, 0.5 mL (Thermo Scientific).

Labelling of Cys-tOmpA with Alexa Fluor 488: Purified Cys-tOmpA at 200 µM in 25 mM Tris-HCl, 6 M guanidine-HCl (Gdn-HCl), pH 7.2, was reduced by incubation with 10 mM DTT at 4 °C for 30 min. The protein was buffer-exchanged into 25 mM Tris-HCl, 6 M Gdn-HCl, pH 7.2 (sparged for 15 min with nitrogen gas) using Zeba spin desalting columns (Thermo-Fisher Scientific, UK). Alexa Fluor 488 C5 maleimide (Thermo-Fisher Scientific, UK) (10 mg/ml dissolved in dimethyl sulfoxide) was immediately added to a final concentration of 2 mM. The total sample volume was 480 µL. The labelling reaction was incubated at 25 °C for 1 h, then left overnight at 4 °C. Free dye was removed by SEC on a Superdex Peptide 10/300 column equilibrated with 6 M Gdn-HCl, 25 mM Tris-HCl, pH 7.2. Fractions were collected every 1 ml and peak protein fractions tested for dye labelling using a Nanodrop 2000 (Thermo-Fisher Scientific, UK), snap-frozen using liquid nitrogen and stored at −80 °C.

Band-shift folding assays of tOmpA in liposomes and nanodiscs: Solutions of 5 µM Alexa Fluor 488-labelled Cys-tOmpA denatured in TBS containing 8 M urea were diluted 5-fold into a 10 µM solution of SurA. This mixture was then immediately diluted 2-fold into BAM-containing nanodiscs or proteoliposomes to initiate folding. A no-nanodisc control was prepared by diluting this mixture into TBS, pH 8.0. The final reaction concentrations were 0.5 µM Alexa Fluor 488-Cys-tOmpA, 5 µM SurA and 0.5 µM BAM nanodiscs/proteoliposomes in TBS, pH 8.0, 0.8 M urea. After folding for 16 h at 25 °C, the reaction was quenched with the addition of 4× SDS-PAGE loading buffer, and the samples loaded onto 15% (w/v) Tris-Tricine SDS-PAGE gels. The gels were imaged using an Alliance Q9 Advanced gel doc (UVITEC, Cambridge, UK) in fluorescence mode under 460-nm light.

OmpT folding activity assays in liposomes and nanodiscs: BAM activity was monitored by the cleavage of a fluorogenic substrate by folded and inserted OmpT, as performed previously^15^. A solution of 1 µM OmpT and 10 μM SurA in TBS, pH 8.0, containing 1.6 M urea was diluted twofold into the BAM-containing proteoliposomes or BAM-containing nanodisc solution in TBS, pH 8.0, containing 2 mM of the fluoropeptide Abz–Ala–Arg–Arg–Ala–Tyr(NO_2_)–NH_2_ (Peptide Synthetics). OmpT–SurA-only and fluoropeptide-only reactions were set up omitting the relevant proteins. The final reaction conditions were 0.5 µM BAM, 0.5 µM OmpT, 5 µM SurA, 1 mM fluoropeptide, TBS, pH 8.0 and 0.8 M urea. Fluorescence of the cleaved peptide was monitored at 430 nm following excitation at 325 nm every 20 s for 5.5 h at 25 °C using a Clariostar plate reader (BMG Labtech GmbH). All assays were carried out in triplicate in a 50-μL final reaction volume, and a buffer-only blank was subtracted from all measurements.

### Electron microscopy

Cryo-EM grid preparation: In total, 300-mesh copper EM grids bearing an R2/1 Quantifoil holey carbon film (EM Sciences) were glow-discharged for 60 s at 10 mA in a Cressington 208 Coating Unit (Cressington Scientific Instruments). In total, 4 µL of a 5 µM BAM-nanodisc sample was applied, grids were blotted with Whatman #1 filter paper, before being vitrified in liquid ethane using a Leica EM-GP plunge freezer (Leica Biosystems GmbH).

Cryo-EM imaging: A total of 15,504 micrographs were recorded from two grids over four sessions using a Titan Krios (Thermo-Fisher) EM operating at 300 keV. Images were recorded on an energy-filtered K2 detector (Gatan Inc.) at a nominal magnification of 130,000× yielding a pixel size of 1.065 Å. The total dose ranged from 35.1 to 50.3 e^−^/Å^2^ over the four datasets. The images were recorded as a series of 20 or 40 frames, resulting in per-frame doses of 1.0–1.8 e^−^/Å^2^. Data collection parameters are shown in Table 1.

**Table 1:** Cryo-EM data collection statistics.

	Consensus structure (EMDB-10247) (PDB 6SMX)			
Data collection and processing				
Magnification	130,000 (nominal)			
Voltage (kV)	300			
	Dataset 1	Dataset 2	Dataset 3	Dataset 4
Electron exposure (e^−^/Å^2^)	41.60	50.37	35.19	37.50
Defocus range (μm)	−1.5 to −3.5	−1.5 to −3.5	−1.5 to 13.0	−1.5 to −3.0
Pixel size (Å)	1.065			
Symmetry imposed	C1			
Final particle images (no.)	208,539			
Map resolution (Å)	6.7 Å			
FSC threshold	FSC = 0.143			

Initial reconstruction: Frames 3–40 or 2–20 of each micrograph movie were motion-corrected, dose weighted and merged using motioncor2^72^. The contrast-transfer function (CTF) for each micrograph was determined using gCTF^73^ on motion-corrected, but non-dose-weighted, micrographs. Particles were selected using Relion3 autopicking^74^ using class averages from a previous reconstruction^15^ filtered to 30 Å as search templates. Individual particles were extracted into 280 × 280 pixels (300-Å^2^) boxes and culled with multiple rounds of 2D and 3D classification in Relion3. The resulting particle stack, containing 208,539 particles, was used to generate the consensus reconstruction. The processing steps and intermediate classes are shown in Supplementary Fig. 10.

Multibody refinement: Masks were created for the top and bottom halves of the consensus reconstruction (Supplementary Fig. 7a) using the volume eraser tool in UCSF Chimera and Relion3 and used for a multibody refinement in Relion3^51^. The principal component analysis identified 6 component motions that accounted for ~85% of the variability in the particles (Supplementary Table 3, Supplementary Movies 1–6). The particles were then further subdivided into classes based on the value of principal components 0 and 1. The individual subclasses were then used to create 3D reconstructions in Relion. All subclass models were independently reconstructed using the consensus model filtered to 60-Å resolution as a starting model (Supplementary Fig. 7b).

Flexible fitting of models to cryo-EM maps: A model was created from BamABCDE pdb entry 5LJO^15^. The C-terminal globular domains of BamC were truncated, leaving only the lasso^40^ region (residues 25–83), resulting in a starting model containing BamA_24–806_, BamB_22–392_, BamC_25–83_, BamD_26–243_ and BamE_24–110_. The starting model was fit into each EM density as a rigid body using UCSF Chimera^75^ and flexibly fit using molecular dynamics flexible fitting^76^.

Analysis of POTRA conformations: A set of Python scripts were used to analyze the relative positions of the POTRA domains in the 16 EM structures and available BamABCDE^13–15^ and BamACDE^12,13^ structures from the literature. The structures were pruned down to a common consensus sequence, the centre of mass (COM) for all Cα atoms in each POTRA calculated and the distances between the COMs plotted in correlation matrices. The POTRA domains were defined as POTRA1 residues 24–92, POTRA2 residues 92–173, POTRA3 residues 173–265, POTRA4 residues 265–347 and POTRA5 residues 350–422. The BAM structures were aligned to each other in different ways depending on which analyses were being performed. For analysis of POTRA5 position, the structures were aligned on the back of the BamA β-barrel opposite the lateral gate (residues 518–767). For analysis of the overall shape of the POTRA corkscrew and the relationship between POTRA4 and POTRA5, the structures were aligned on POTRA5. For analysis of the additional POTRA relationships, the structures were aligned on the previous POTRA domain, i.e., POTRA4 for analysis of POTRA4–POTRA3, POTRA3 for analysis of POTRA3–POTRA2 and so forth. All scripts used for sequence normalization, alignment, COM calculation and transformation of the relative position of the POTRAs are available at https://github.com/attamatti/movement_analysis and https://github.com/attamatti/COM_analysis.

Comparison of the detergent and nanodisc BAM maps: Models of the detergent-solubilized BAM complex, PDB 5LJO^15^ and the consensus BAM-nanodisc structure, were fit into their respective maps (EMDB-4061^15^ and the consensus map, respectively) in UCSF Chimera^75^. All densities within 4 Å of the fitted structures were selected and the remaining density subtracted to remove the density from the micelle or nanodisc. The subtracted maps were aligned in Chimera and the overall cross-correlation calculated with Chimera and FSC between the aligned maps calculated using relion_image_handler^74^.

Fitting MSP structures to the nanodisc BAM map: For the analyses in Supplementary Figs. 5 and 15 an MSP1D1 nanodisc model was created using CHARMM-GUI^77,78^, the lipids removed and the MSPs manually fit the EM density. It should be noted that the overall orientation of the two MSPs and locations of the termini relative to BAM and each other is the best guess based on the density. The MSP model was then flexibly fit using MDFF^76^.

### MD simulations

Set-up and running of MD simulations: All-atom 1-μs MD simulations were performed in explicit solvent using GROMACS 2016.4^79^ and the CHARMM36 force field^80^. Simulation systems were built with CHARMM-GUI^77,78^ and made use of the Nanodisc Builder input generator^78^. The MSP1D1 nanodiscs in each system contained three different lipid types: 1-palmitoyl(16:0)-2-vacenoyl (18:1 *cis*-11)-phosphatidylethanolamine (PVPE), 1-palmitoyl(16:0)-2-vacenoyl (18:1 *cis*-11)-phosphatidylglycerol (PVPG) and 1,1′-palmitoyl-2,2′-vacenoyl cardiolipin with a net charge of −2*e* (PVCL2). The molar ratio was ~70:25:5 PVPE:PVPG:PVCL to approximate the composition of the *E. coli* polar lipid extract used experimentally. Simulations of BAM in a MSP1D1 nanodisc used the BamABCDE complex (PDB: 5LJO^15^) as a starting structure, with missing N- and C-terminal residues built-in Chimera^75^. Lipidation of the N-terminal Cys residues of BamB–E was performed in CHARMM-GUI^77^. For the simulation of the OmpA β-barrel domain (tOmpA), the starting structure was taken from PDB: 1QJP^47^, with mutated residues in the structure replaced with wild-type residues and missing residues in the loops built-in using MODELLER^81^. Full details of the molecular contents of each simulation are listed in Supplementary Table 2.

Systems were minimized (5000 steps) with protein backbone, protein sidechain and lipid position restraints of 400, 40 and 1000 kJ mol^−1^ nm^−2^, respectively, followed by equilibration for a total of 375 ps during which these restraints were gradually released. In the BAM complex simulation, a further equilibration step of 30 ns was performed to allow the lipoprotein acyl chain tails of BamB–E to equilibrate in the nanodisc bilayer. During this step, a position restraint of 1000 kJ mol^−1^ nm^−2^ was applied to the protein backbone of BamA–E. A force-switch function was used to smoothly switch off van der Waals interactions between 1.0 and 1.2 nm^82^, and the particle-mesh Ewald method was used to calculate long-range electrostatic interactions^83^. In all systems, the pressure was maintained using a Parrinello–Rahman barostat^84^ and the temperature was maintained using a Nose–Hoover thermostat^85^. The pressure and temperature of the systems was 1 bar and 303.15 K, respectively, and the timestep was 2 fs.

Analysis of membrane thickness and MSP planarity in MD simulations: Protein and lipid atom coordinates were extracted every 100 frames of the MD simulation using the GROMACs ‘*gmx trjconv’* command, yielding 250 individual structures for each simulation. A series of python scripts were written to analyze the membrane thickness and nanodisc planarity for each frame separately and the resulting data compiled. For membrane thickness, the phosphate atom in each lipid was used to define its position. A plane was fit to all phosphate atoms and each lipid was assigned to either the top or bottom leaflet, based on its location relative to this plane. Individual planes were then fit to each of the leaflets and the average of these used to determine a more accurate central plane. The coordinates of each phosphate atom were then projected onto the average central plane and the absolute distance between the phosphate and its projection on the central plane used to determine its height. The area inside the nanodisc on the central plane was sampled on a 1 × 1-Å grid and the height of any given point on the grid calculated as the mean height of the three closest lipid headgroups. This calculation was performed separately for the upper and lower leaflets, and the sum used to determine the bilayer thickness at the given point. Mean bilayer thickness and standard deviations are reported over all 250 structures.

For MSP planarity, a similar but separate analysis was performed. The upper and lower MSPs were each analyzed separately. For each, a plane was fit to the Cα atoms of the MSP protein and the coordinates of each Cα (*x*, *y*, *z*) projected onto the plane (*x*_*p*_, *y*_*p*_, *z*_*p*_). The sum deviation from planarity for each Cα was then reported as:

$${\rm{log}}\left( {\mathop {\sum}\limits_{n = 1}^{251} {\sqrt {( {x - x_p} )^2 + ( {y - y_p} )^2 + ( {z - z_p} )^2} } } \right).$$

All analyses and fitting were performed using numpy^86^ and scipy^87^. Scripts used for these analyses are available at https://github.com/attamatti/nanodisc_lipid_analysis.

### smFRET experiments

Preparation of labelled BAM: Dye-containing solutions were protected from light at all times to reduce photobleaching. In total, 1 mg of lyophilized Alexa Fluor 488 maleimide and DyLight 594 maleimide dyes were reconstituted in DMSO to a stock concentration of 10 mM. About 200 μL of ~95 μM BAM(R127C/N520C/C690S/C700S) was buffer-exchanged using a 0.5-ml ZebaSpin 7 k MWCO desalting column into reducing labelling buffer (10 mM DTT, 0.05% (w/v) DDM, 1 mM EDTA, 150 mM NaCl and 50 mM Tris-HCl, pH 7.6) and allowed to reduce for 45 min at 4 °C. The sample was then buffer-exchanged into nitrogen-sparged labelling buffer (as above, but lacking reducing agent). The molar excess of each dye over BAM was varied until 50:50 labelling of BAM with each dye was achieved; the best conditions were found to be an ~1.75-times molar excess of Alexa Fluor 488, and an ~8.8-times molar excess of DyLight 594. Dyes were pre-mixed and added to BAM(R127C/N520C/C690S/C700S), 10 μL at a time, in quick succession, while mixing. Once the full amount of dye had been added, the sample was put on a roller and allowed to react at room temperature (~23 °C) for 1 h. This reaction was quenched with a 100-fold molar excess of DTT over the dyes. The volume was then adjusted with labelling buffer to 200 μL before free dye was removed using a Superdex 200 10/30 GL column pre-equilibrated with running buffer (0.05% (w/v) DDM, 150 mM NaCl and 20 mM Tris-HCl, pH 8.0). The separation was performed at 0.5 ml min^−1^ with constant monitoring at 280, 495 and 590 nm. In total, 0.5-ml fractions corresponding to the double-labelled protein were pooled and concentrated to ~500 μL in a 50-kDa MWCO VivaSpin 20 centrifugal concentrator. Protein concentration and labelling stoichiometry were determined using UV spectroscopy (NanoDrop 2000) with A_280_ corrections to subtract the additional absorbance at 280 nm from the dyes themselves. This gave a stoichiometry of 0.48 for Alexa Fluor 488, 0.52 for DyLight 594. In total, 3-μL aliquots were snap-frozen in liquid nitrogen and stored at −80 °C.

smFRET experiments: smFRET was performed using a custom-built set-up for μs alternating-laser excitation^88^. Laser wavelengths and powers used were 488 nm, 140 μW and 594 nm, 120 μW. The laser alternation period was 40 μs (duty cycle of 40%). Samples of labelled BAM in MSP1D1 nanodiscs were prepared on the day of use from concentrated stocks (see above) and kept on ice in the dark while not in use. All measurements were made at room temperature (~23 °C), the same temperature at which the cryo-EM grids were prepared. A sample of labelled BAM (100 µL, 20 mM Tris-HCl, pH 8.0, 150 mM NaCl, 20 pM) was added atop a coverslip set on the objective. A camera was used to monitor the distance of the focal plane from the coverslip and the objective height adjusted using a piezocontroller (Piezosystem Jena) to 20 μm above the surface of the coverslip. Data acquisition was performed in 3 × 10-min runs with the fresh sample prepared after every third collection to control for protein aggregation, adherence to the coverslip and changes in osmolarity from evaporation. Evaporation was further minimized by using a coverslip lid. Data were collected using the LabVIEW graphical environment (LabVIEW 7.1 Professional Development System for Windows, National Instruments, Austin, TX)^89^. Separate photon streams were then converted and stored in an open-file format for timestamp-based single-molecule fluorescence experiments (Photon-HDF5), which is compatible with many recent data-processing environments^90^. The data from each 10-min acquisition were merged prior to subsequent analysis. Fluorescence bursts were analyzed using custom Python 2.7 scripts^91^ and made use of FRETBursts, an open-source toolkit for analysis of freely-diffusing smFRET bursts^92^. Functions from the FRETBursts package were used to estimate the background signal as a function of time, identify and remove artefacts due to photophysical effects such as blinking and provide an optimal signal-to-noise ratio. To obtain bursts, an initial lower threshold was set of 20 photons per burst. Three correction parameters were applied as described previously^91^: γ-factor (to account for differences in the efficiency of excitation of each dye), donor leakage into the acceptor channel and acceptor direct excitation by the donor excitation laser. In order to remove bursts arising from single-labelled proteins, burst data were filtered using ALEX-2CDE, yielding bursts with a Gaussian distribution of S values in a narrow range of dye stoichiometry (S within 0.25–0.75)^93^. A final lower-threshold filter of three photons in the acceptor channel during donor excitation was then applied. In total, 8582 bursts were collected after all filters had been applied. Filtered bursts were then analyzed by recurrence analysis^52^ performed using previously described Python (2.7 or 3.7) scripts^91^. Briefly, recurrence analysis employs extremely diluted samples, where the probability that a molecule will return to the confocal volume within a short temporal window is greater than the probability that a new molecule will be detected. The longest usable recurrence time is related by concentration and diffusion time of the observed objects, and can be set based on the recurrence probability. The recurrence probability of single molecules was estimated using a correlative method^49^. Bursts from different and non-interacting molecules are expected to be uncorrelated, whereas, bursts originating from the same molecule should be correlated and the ‘same molecule’ recurrence probability *P*_same_(*t*) can be calculated:

$$P_{\rm{same}}\left( t \right) = 1 - 1/g\left( t \right),$$

where *g*(*t*) is the burst-time autocorrelation function of all detected bursts. From a fit to the data, we determined for each burst pair the probability that it originated from the same, recurring molecule, and calculated the average *P*_same_ for a subset of bursts by averaging over all corresponding burst pairs. The burst pairs were plotted and fitted to a 2D Kernel Density Estimator^94^ with a bandwidth of 0.012 using the Seaborn and Matplotlib^95^ packages. Visualizations of the available volumes for FRET dyes attached at positions R127 and N520 of BamA were generated using the FRET Positioning and Screening (FPS) software with dye linker lengths and radii parameters as suggested in the FPS manual for the FRET dyes used^53^. Predicted mean E_FRET_ values for each of the 16 cryo-EM-derived structures were calculated using an *R*_0_ of 60 Å from distance distributions between the labels generated using the PyMOL plugin ‘MtsslWizard’^54^ and fitted to single Gaussian distributions for comparison with experimentally measured values (Supplementary Table 5).

### Statistics and reproducibility

smFRET data were collected using a single preparation of nanodiscs. Each sample was diluted immediately prior to measurement and run in triplicate, for a total of 30 runs and 10 individual preparative dilutions. The data object comprising all of the recovered bursts that contained more than 30 photons (*N* = 29,553) was subjected to refinement as detailed in the Methods section, resulting in *N* = 8582 after all spurious bursts had been removed.

Fluorescence-based activity assays were conducted in triplicate. Each data series in Fig. 2a shows the mean value from the three replicates with the minimum and maximum value for the datapoint.

### Analysis of crystal-packing contacts

The closed lateral gate BamABCDE X-ray crystal structures (PDBs: 5D0O^13^ and 5AYW^14^) are nearly identical (Cα RMSD 1.88 Å over the entire structure) and have the same unit-cell dimensions and crystallographic symmetry, but crystal packing differs between the two structures. Furthermore, the asymmetric unit of 5D0Q^13^ contains two copies of BamACDE, with different structures and crystal contacts. These were analyzed separately. A 3 × 3 unit-cell region around each structure was generated using UCSF chimaera, and all atoms with a van der Waals radius overlap of greater than 0.4 Å identified as potential contacts. Python scripts were then used to analyze the lists of overlaps to identify potential salt bridges, defined as Lys/Arg sidechain amides contacting Asp/Glu sidechain carboxylic acids, and potential hydrophobic interactions, defined as carbon atoms with the above >0.4-Å van der Waals radius overlap. Hydrogen bonds were identified using UCSF Chimera’s ‘findHbonds’ feature using default parameters. Scripts used for crystal-packing analysis are available at https://githib.com/attamati/crystal_packing_analysis.

### Reporting summary

Further information on research design is available in the Nature Research Reporting Summary linked to this article.

## Supplementary information

Supplementary Information

Description of Additional Supplementary Files

Supplementary Movie 1

Supplementary Movie 2

Supplementary Movie 3

Supplementary Movie 4

Supplementary Movie 5

Supplementary Movie 6

Reporting Summary

## Data Availability

The cryo-EM map and fitted model for the consensus structure are available from the PDB and EMDB accession numbers 6SMX and EMD-10247. The 16 maps and models 0–1 through 1–8 from the ensemble analysis are available as PDB entries 6SNO, 6SN2, 6SN3, 6SN4, 6SN5, 6SN7, 6SN8, 6SN9, 6SOA, 6SO7, 6SO8, 6SOB, 6SOC, 6SOG, 6SOH and 2SOJ, and EMDB entries 10,248; 10,249; 10,250; 10,251; 10,252; 10,253; 10,254; 10,255; 10,270; 10,268; 10,269; 10,271; 10,272; 10,274; 10,275; 10,276, respectively. The original 3,107,582 autopicked particles from which the dataset for this paper was drawn are available via the EMPIAR database (Deposition code 680). Any remaining info can be obtained from the corresponding author upon reasonable request.

## Code availability

All scripts used for sequence normalization, alignment, centre-of-mass calculation and transformation of the relative position of the POTRAs are freely available: https://github.com/attamatti/movement_analysis and https://github.com/attamatti/COM_analysis.
