# Supplementary material for: Distortion of the bilayer and dynamics of the BAM complex in lipid nanodiscs
Source: Commun Biol. 2020 Dec 14;3:766. doi: 10.1038/s42003-020-01419-w (PMC7736308; doi:10.1038/s42003-020-01419-w)
Supplement: Supplementary file 1 — Supplementary Information [file 42003_2020_1419_MOESM1_ESM.pdf]

SUPPLEMENTARY INFORMATION

Supplementary Table 1: Survey of membrane protein lipid nanodisc cryoEM structures

protein	Description	Type	Oligomer	Helices/strands per monomer	Resolution (Å)	Applied symmetry	Nanodisc	Diameter (Å)	Lipid	Structured lipid resolved[+]	Distortion reported
TRPV1[1]	unliganded	helical	6	4	3.2	C4	MSP2N2	150[a]/165[b]	soybean polar lipid extract	yes	No
TRPV1[1]	agonist-bound	helical	6	4	2.9	C4	MSP2N2	150[a]/165[b]	soybean polar lipid extract	yes	No
TRPV1[1]	antagonist-bound	helical	6	4	3.4	C4	MSP2N2	150[a]/165[b]	soybean polar lipid extract	no	No
PKD2[2]		helical	6	4	3	C4	MSP2N2	150[a]/165[b]	soybean polar lipid extract	no	No
RyR1[3]	+ ryanodine	helical	6	4	7.3	C4	MSP1E3D1	121[a]	soybean polar lipid extract	no	No
RyR1[3]	human 'primed'	helical	6	4	6.2	C4	MSP1E3D1	121[a]	soybean polar lipid extract	no	No
Kv chimera[4]		helical	6	4	3.3	C4	MSP1E3D1	121[a]	POPE:POPG 3:1	no	No
Rhodopsin[5]		helical	2	7	4.7	C2	MSP1E3D1	121[a]	POPC	no	No
α1β2/3γ2 GABAA[6]		helical	5	4	3.2	C1	MSP2N2	150[a]/165[b]	POPC + bovine brain extract	no	No
INX-6[7]		helical	8	4	3.8	C8	MSP2N2	150[a]/165[b]	POPC	no	No
INX-6ΔN[7]		helical	8	4	4	C8	MSP2N2	150[a]/165[b]	POPC	no	No
ABCG2[8]		helical	2	6	3.1	C2	MSP1D1	95[a]/97[b]	brain polar lipid extract	yes	No
ABCG2[8]	+ inhibiter + FAb	helical	2	6	3.6	C1	MSP1D1	95[a]/97[b]	brain polar lipid extract	yes	No
ABCG2[8]	+ inhibiter	helical	2	6	3.6	C1	MSP1D1	95[a]/97[b]	brain polar lipid extract	yes	No
RyR1[9]	rabbit "open"	helical	6	4	8.5	C4	MSP1E3D1	121[a]	POPC	no	No
RyR1[9]	rabbit "closed	helical	6	4	6.1	C4	MSP1E3D1	121[a]	POPC	no	No
ribosome–SecYE[10]		helical	1/1	10 /3	7.1	C1	apoA1 Δ1-34	variable	E. coli polar lipid extract	no	No
TRPML[11]	closed I	helical	6	4	3.64	C4	MSP1	97[a]/98[b]	POPC:POPG:POPE 3:1:1	yes	No
TRPML[11]	closed II	helical	6	4	3.75	C4	MSP1	97[a]/98[b]	POPC:POPG:POPE 3:1:1	yes	No
NOMPC[12]		helical	6	4	3.55	C4	MSP2N2	150[a]/165[b]	soybean polar lipid extract	yes	No
hTRPV6[13]	closed	helical	6	4	3.6	C4	CNW11	110[c]	soybean polar lipid extract	yes	No
rTRPV6*[13]	closed	helical	6	4	3.9	C4	CNW11	110[c]	soybean polar lipid extract	no	No
TMEM16F[14]	class1	helical	2	10	3.2	C1	MSP2N2	150[a]/165[b]	SoyPC:POPC:POPE:POPS 6:3:1:1	no	Yes
TMEM16F[14]	class2	helical	2	10	3.3	C1	MSP2N2	150[a]/165[b]	SoyPC:POPC:POPE:POPS 6:3:1:1	no	Yes
Connexin-46/50[15]		helical	6	4	1.9	AD6	MSP1E1	104[a]/106[b]	DMPC	yes	No
nhTMEM16[16]		helical	2	10	3.79	C1	MSP2N2	150[a]/165[b]	POPC:POPG 7:3	no	Yes
SAM complex[17]		barrel	1	16	3.4	C1	MSP1E3D1	121[a]	DOPE:DOPC 1:1	no	no
BAM complex		barrel	1	16	6.7	C1	MSP1D1	95[a]/97[b]	E. coli polar lipid extract	no	yes

[a] Stokes hydrodynamic diameter, determined by size-exclusion chromatography[18] [b] diameter determined by SAXS[18] [c] diameter based on image measurements[9]. [+] If any number of structured lipids were reported as visible the map was counted as having structured lipids.

Supplementary Table 2. Molecular composition used in molecular dynamics simulations

Protein (PDB)	BAM (5LJO)[19]	tOmpA (1QJP)[20]	None
MSP1D1 copies	2	2	2
Lipids			
PVPE[a]	112	112	140
PVPG[b]	40	40	50
PVCL[c]	8	8	10
Other molecules			
Na^+	739	286	297
Cl^-	620	217	217
H_2O	208,328	78,300	78,127
Total atoms	679,426	265,176	267,349
Box size (nm)	18.9^3	13.8^3	13.8^3

[a] 1-palmitoyl(16:0)-2-vacenoyl (18:1 cis-11)-phosphatidylethanolamine
[b] 1-palmitoyl(16:0)-2-vacenoyl (18:1 *cis*-11)-phosphatidylglycerol
[c] 1,1′-palmitoyl-2,2′-vacenoyl cardiolipin

Supplementary Table 3. Eigenvectors describing relative motions of the two masked halves of BAM for the first 6 principal components

Principal component	% variability	Body 1 - Top					
		Rot ˚	Tilt ˚	Psi˚	x Å	y Å	z Å
0	19.89	0.03	-0.01	0.04	0.00	-0.01	0.01
1	17.05	0.60	0.65	-0.46	0.00	0.02	-0.01
2	13.65	-0.01	0.06	-0.02	-0.03	0.00	-0.04
3	12.04	0.69	-0.31	0.50	-0.01	0.04	-0.03
4	11.27	-0.03	-0.50	-0.69	-0.04	-0.03	-0.01
5	10.99	0.40	-0.47	-0.24	0.00	0.04	-0.01
Principal component	% variability	Body 2 - Bottom					
		Rot ˚	Tilt ˚	Psi˚	x Å	y Å	z Å
0	19.89	-0.36	-0.04	0.92	0.00	0.03	-0.13
1	17.05	0.06	-0.01	0.03	-0.01	0.00	0.01
2	13.65	-0.49	0.85	-0.15	0.08	-0.06	-0.03
3	12.04	0.32	0.24	0.08	0.02	0.05	-0.05
4	11.27	0.40	0.28	0.19	0.01	0.04	-0.05
5	10.99	-0.60	-0.36	-0.24	0.02	-0.10	0.03

Supplementary Table 4. Statistics for classes separated on Eigenvalues of components 0 or 1.

	Eigenvalue component 0					Reconstruction
Class	Min	Max	Mean	StDev	# particles	Resolution (Å)
0-1	-15	-8	-10.07	1.66	11134	10.8
0-2	-8	-5	-6.31	0.85	20584	9.5
0-3	-5	-2	-3.37	0.86	37910	8.4
0-4	-2	-0.5	-1.23	0.43	25541	9.5
0-5	-0.5	0.5	0.00	0.29	18182	9.8
0-6	0.5	2	1.23	0.43	25798	8.9
0-7	2	5	3.37	0.86	37529	8.4
0-8	5	8	6.31	0.85	20115	9.8
0-9	8	15	10.1	1.67	11377	10.8
	Eigenvalue component 1					Reconstruction
Class	Min	Max	Mean	StDev	# particles	Resolution (Å)
1-2	-8	-5	-6.19	0.82	13078	10.5
1-3	-5	-3	-3.89	0.57	21840	9.8
1-4	-3	-0.5	-1.63	0.71	51609	8.5
1-5	-0.5	0.5	0.00	0.29	26388	9.0
1-6	0.5	3	1.63	0.71	53634	8.3
1-7	3	5	3.87	0.57	21982	9.5
1-8	5	8	6.17	0.82	12708	10.4

Supplementary Table 5. Fit data for E_{FRET} of cryo-EM derived models

model	weight	centre	sigma	fwhm	amplitude	red.χ^2	AV3
0-1	0.244	0.173	0.055	0.129	0.172	0.006	N/A
0-2	0.407	0.185	0.058	0.136	0.296	0.011	0.14
0-3	0.962	0.158	0.052	0.122	0.631	0.097	0.13
0-4	0.492	0.181	0.061	0.143	0.375	0.024	0.14
0-5	1	0.188	0.062	0.145	0.763	0.089	0.14
0-6	0.409	0.178	0.061	0.143	0.309	0.018	0.13
0-7	0.237	0.189	0.064	0.151	0.191	0.005	0.14
0-8	0.208	0.200	0.066	0.156	0.173	0.004	0.14
0-9	0.384	0.248	0.088	0.208	0.435	0.019	0.2
1-0	0.707	0.205	0.071	0.166	0.625	0.053	0.18
1-1	0.476	0.192	0.064	0.151	0.385	0.022	0.16
1-2	0.339	0.201	0.072	0.169	0.310	0.015	0.16
1-3	0.481	0.193	0.066	0.156	0.394	0.029	0.16
1-4	0.700	0.205	0.071	0.167	0.634	0.047	0.15
1-5	0.375	0.199	0.068	0.160	0.326	0.013	0.14
1-6	0.212	0.200	0.066	0.155	0.172	0.005	0.18

Different modelling approaches yield slightly different results. Although the mtssl wizard returned values that matched experiment better across the board, the FPS modelling approach afforded a better match for the experimental data with model (0-3 red) but could not resolve a value for one of the models (0-1 N/A). The models used were built from atomic coordinates of the protein derived from the flexibly fitted cryo-EM models and inserted by alignment and replacement into an MD frame to afford the position of lipid molecules. Thus, we propose that the exact locations of lipid molecules selected in this way can occlude the attachment position of the dye in some cases and potentially influence the exact value returned from the modelling approaches employed. Nevertheless, in the physical world, the dyes are attached prior to incorporation into the lipid environment so conformations that are perhaps compromised *in silico* will still occur. We were satisfied with the agreement between experiment and the majority of the modelled values and hence do not feel that an exhaustive search of the conformational space afforded by the simulation to optimise the locations of lipid molecules prior to modelling of FRET behaviour is warranted

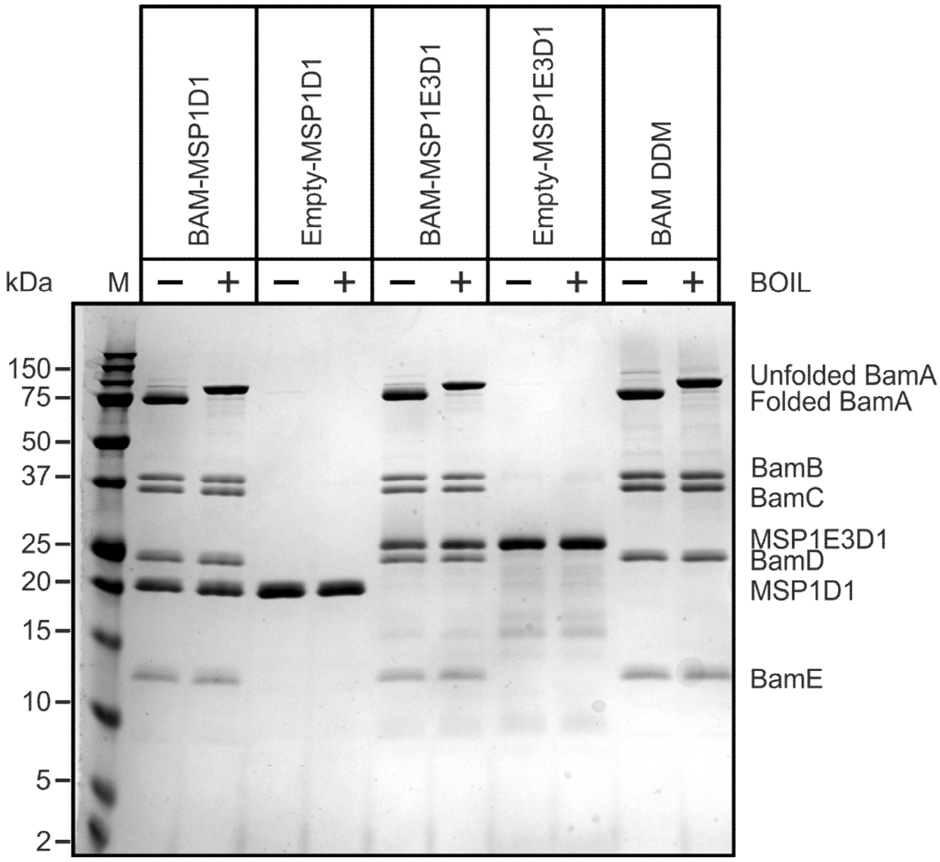

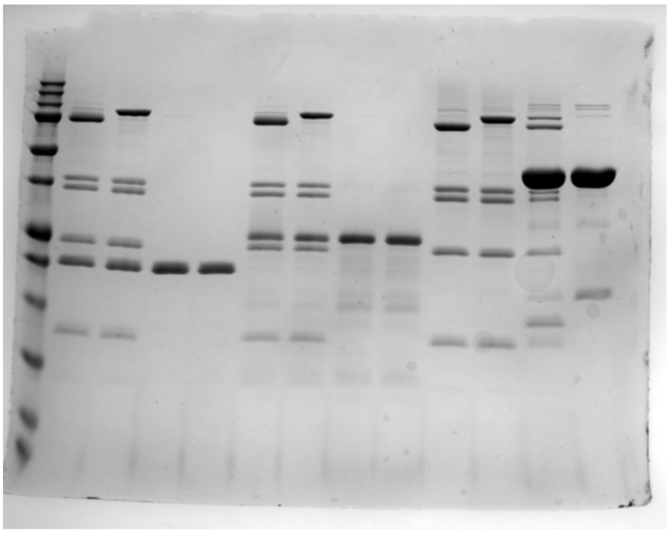

UNCROPPED GEL

Supplementary Figure 1: Characterisation of BAM-MSP1D1 or MSP1E3D1 nanodiscs. SDS-PAGE gel of detergent (DDM) solubilized BAM, and BAM-containing or empty MSP1D1 and MSP1E3D1 nanodiscs. All five BAM subunits are present in the detergent solubilized and nanodisc preparations. BamA shows a band shift after heating in both preparations, confirming that the BamA β-barrel is folded in DDM and in both nanodiscs.

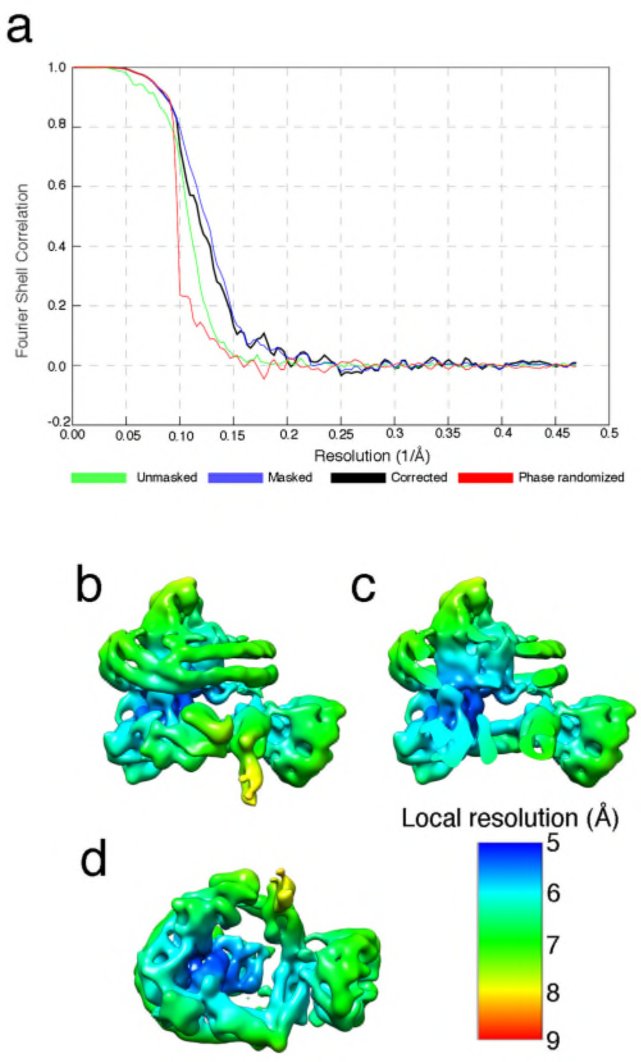

Supplementary Figure 2. Fourier shell correlation (FSC) and local resolution of the consensus structure. (a) FSC of the two independent half maps from the reconstruction of the consensus structure showing masked (red), unmasked (green), corrected (black), and phase randomized (red) maps. Local resolution of the consensus map viewed from the side (b), cut away to highlight the lateral gate (c), and from the bottom (periplasmic) face (d).

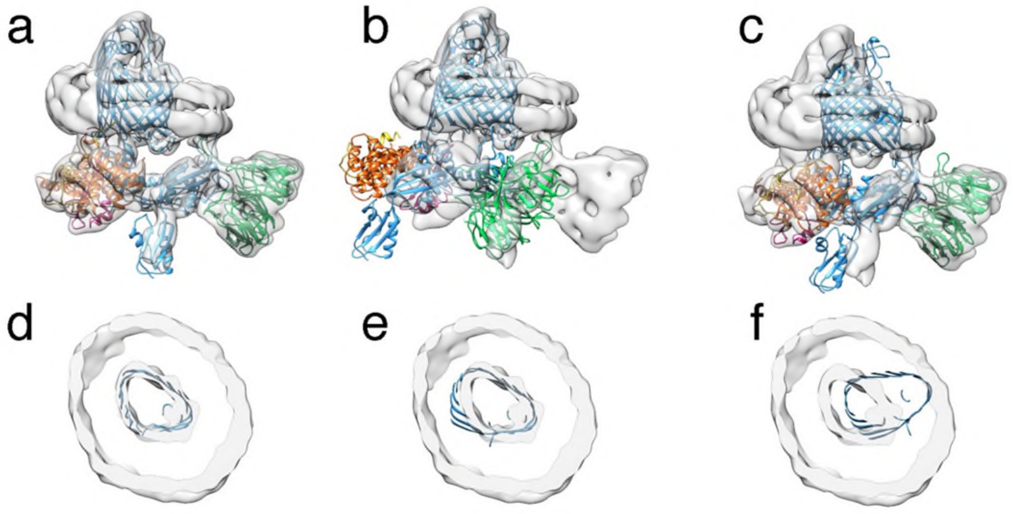

Supplementary Figure 3. Fitting the lateral open and lateral closed structures to the BAM-nanodisc consensus map . (a) Detergent solubilized BamABCDE in the lateral open conformation determined by cryoEM (PDB: 5LJO[19]) globally fit. (b) Detergent solubilized BamABCDE in the lateral closed conformation determined by X-ray crystallography (PDB: 5D0O[21]) fit using the BamA β-barrel only (residues 420-809). (c) The lateral closed conformation globally fit. (d-f) Cut away of the top (cytoplasmic face) of the complex showing the BamA β-barrel shape in the (d) globally fit lateral open structure (5LJO) and the (e) β-barrel fit and (f) globally fit lateral closed structure (5D0O).

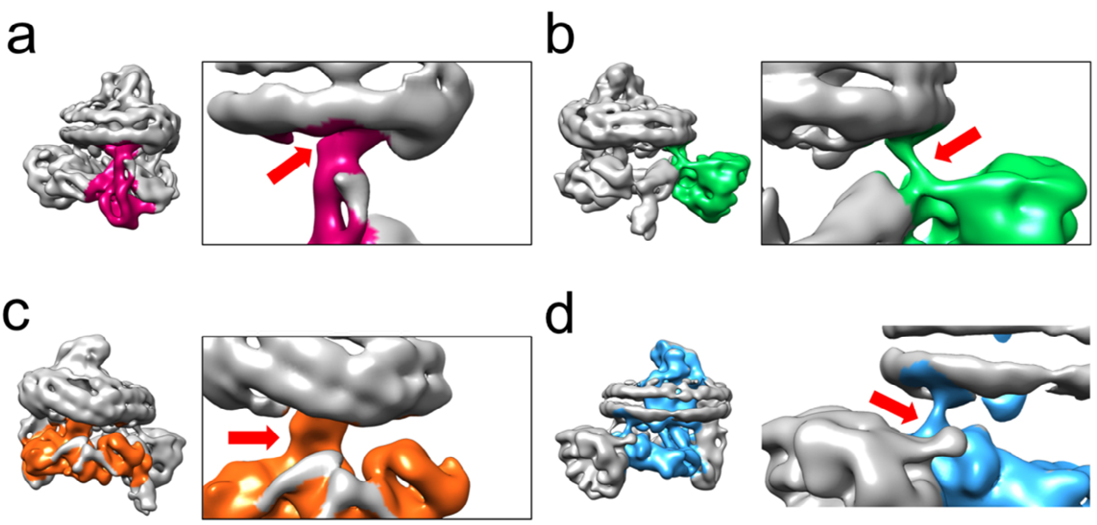

Supplementary Figure 4. Interactions with the lipid bilayer visualized in the BAM-nanodisc structure. a) The N-terminal region of BamE (pink), containing its lipid anchor (arrow, inset), b) the N-terminal region of BamB (green) containing its lipid anchor (arrow, inset), c) a 3_{10} helix in BamD (orange) composed of $BamD_{123\text{-}129}$ (arrow, inset) and d) a loop on POTRA3 (cyan) containing $BamA_{200\text{-}213}$ (arrow, inset). In all images intact BAM in MSP1D1 is shown with the membrane-bound region expanded and inset.

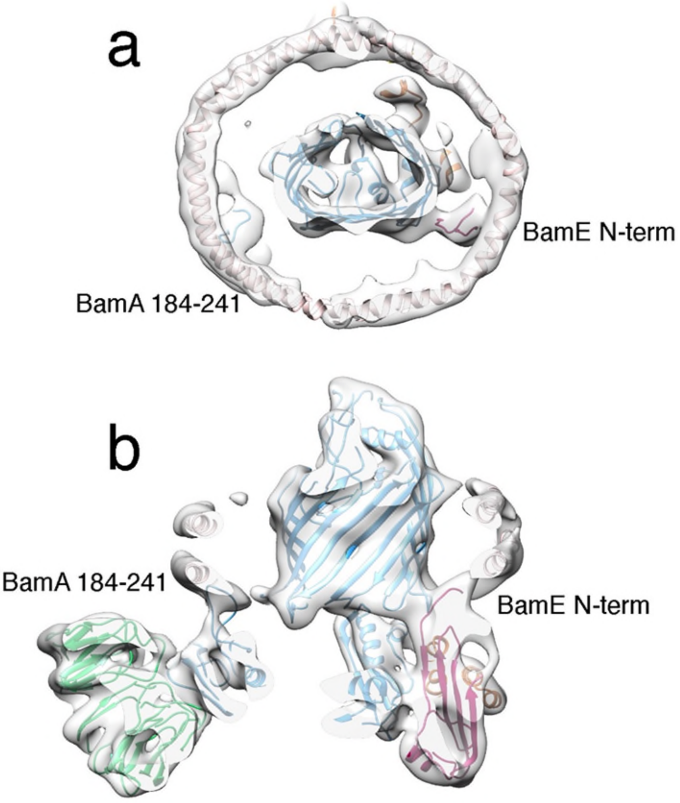

Supplementary Figure 5. Possible contacts between BAM and the nanodisc MSPs. (a) Top view and (b) sideview of the density of the consensus map with the fit BAM and MSPs. The density is cut away to reveal two regions where a loop of BamA (left) and the N-terminus of BamE (right) could possibly make contact with the nanodisc MSPs.

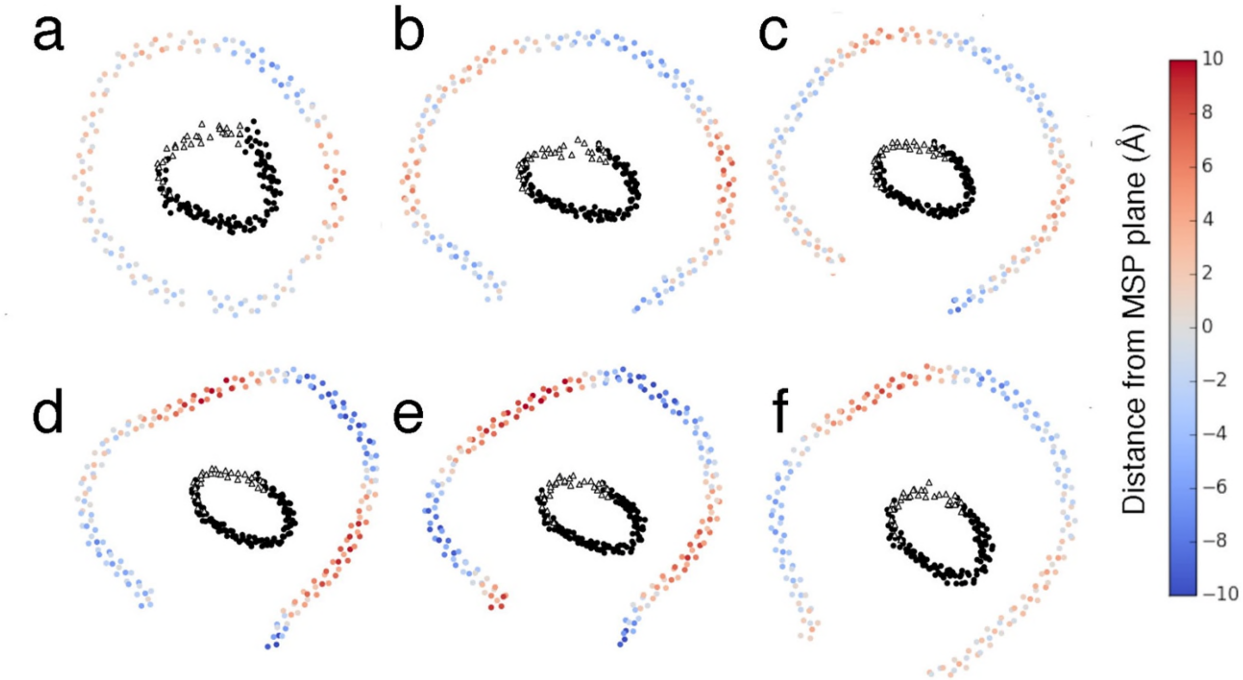

Supplementary Figure 6. Comparison of MSP1D1 in the EM map and MD simulation. Deviation of the Cα atoms of MSP1D1 from the overall plane of the nanodisc in (a) model MSP1D1 flexibly fit into the EM density of the consensus map, and MSP1D1 of the MD simulation at (b) 200 ns, (c) 400 ns, (d) 600 ns, (e) 800 ns and (f) 1 μs. In all panels the Cα atoms of β-strands 3-14 of BamA are designated with black dots and β-strands 1,2,15, and 16 (the lateral gate) are designated with white triangles.

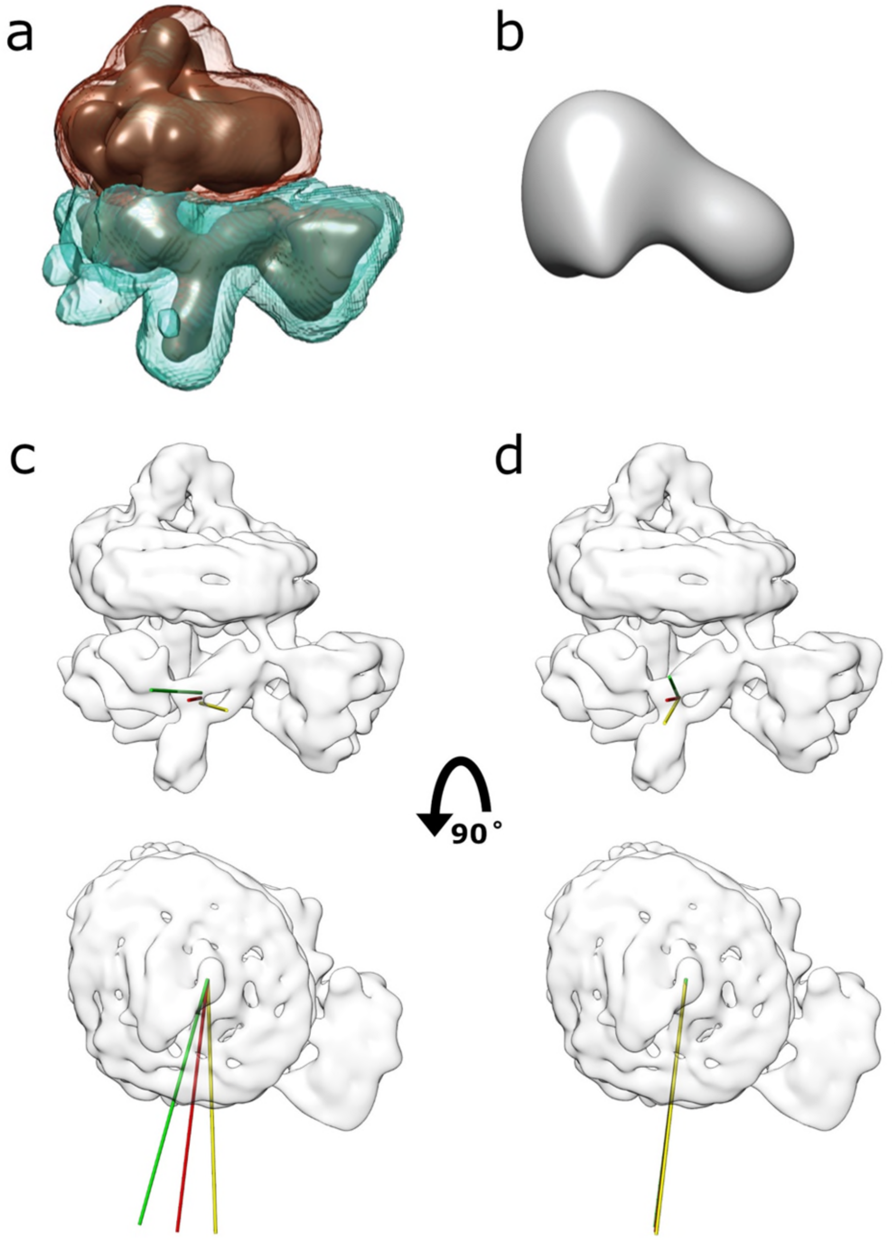

Supplementary Figure 7. Inputs used for refinement of classes separated on eigenvectors. a) Masks (brown/cyan) used for multibody refinement of the BAM consensus dataset, and b) the starting model each subset was refined against. c) and d) Lines overlaid on the consensus structure to illustrate the ranges of component motions 0 (c) and 1 (d). In each the red line is an arbitrary vector aligned on the bottom half structure with a value of 0 for the component motion eigenvector. Positive and negative extremes of the eigen vector are represented by yellow and green lines respectively.

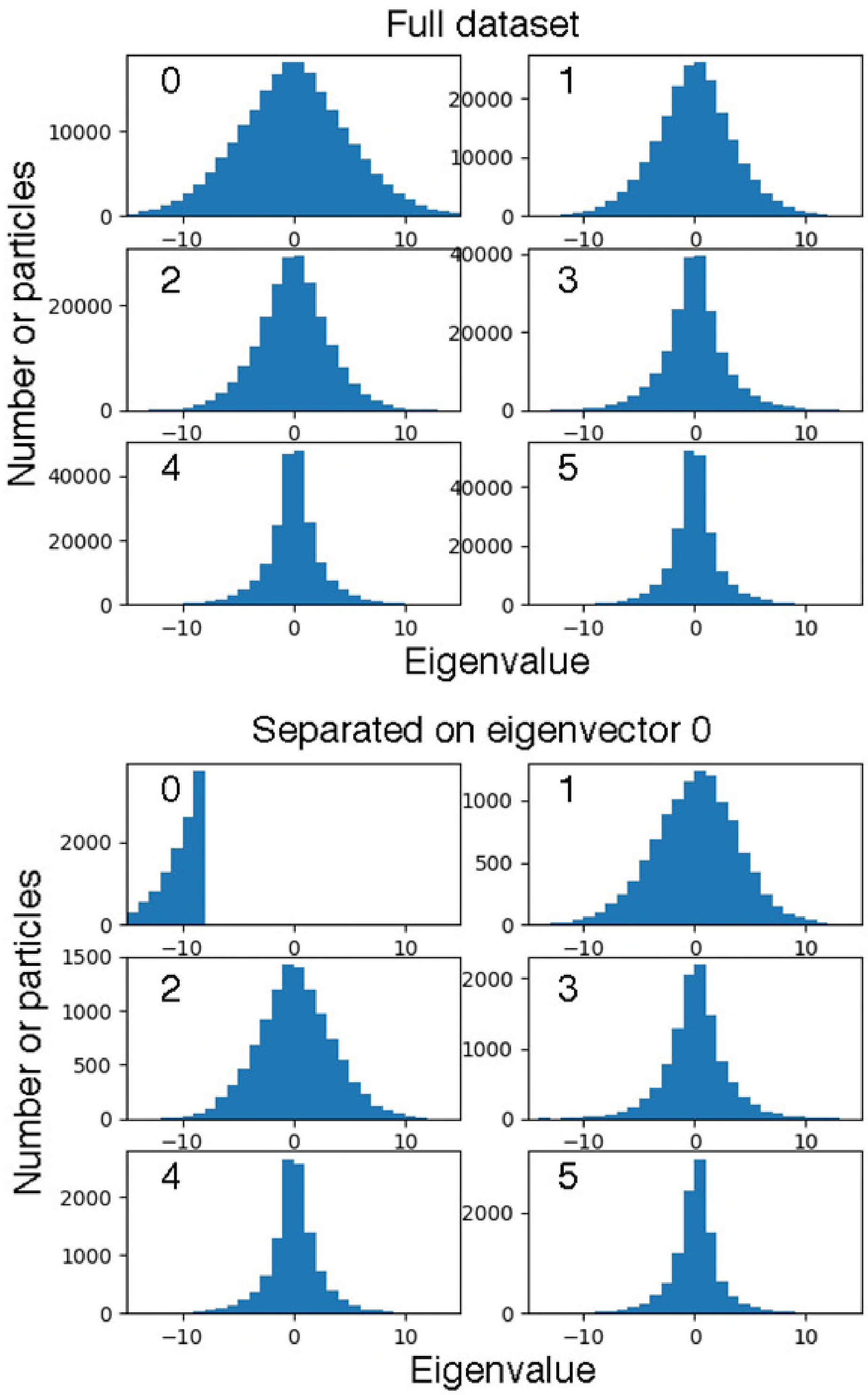

Supplementary Figure 8a. Eigenvalue distributions for class 0-1. Comparison of Eigenvalue distributions for the full dataset (top) and subclass 0-1 (bottom). Each subplot shows distributions of values for eigenvectors noted in the top left of the subplot.

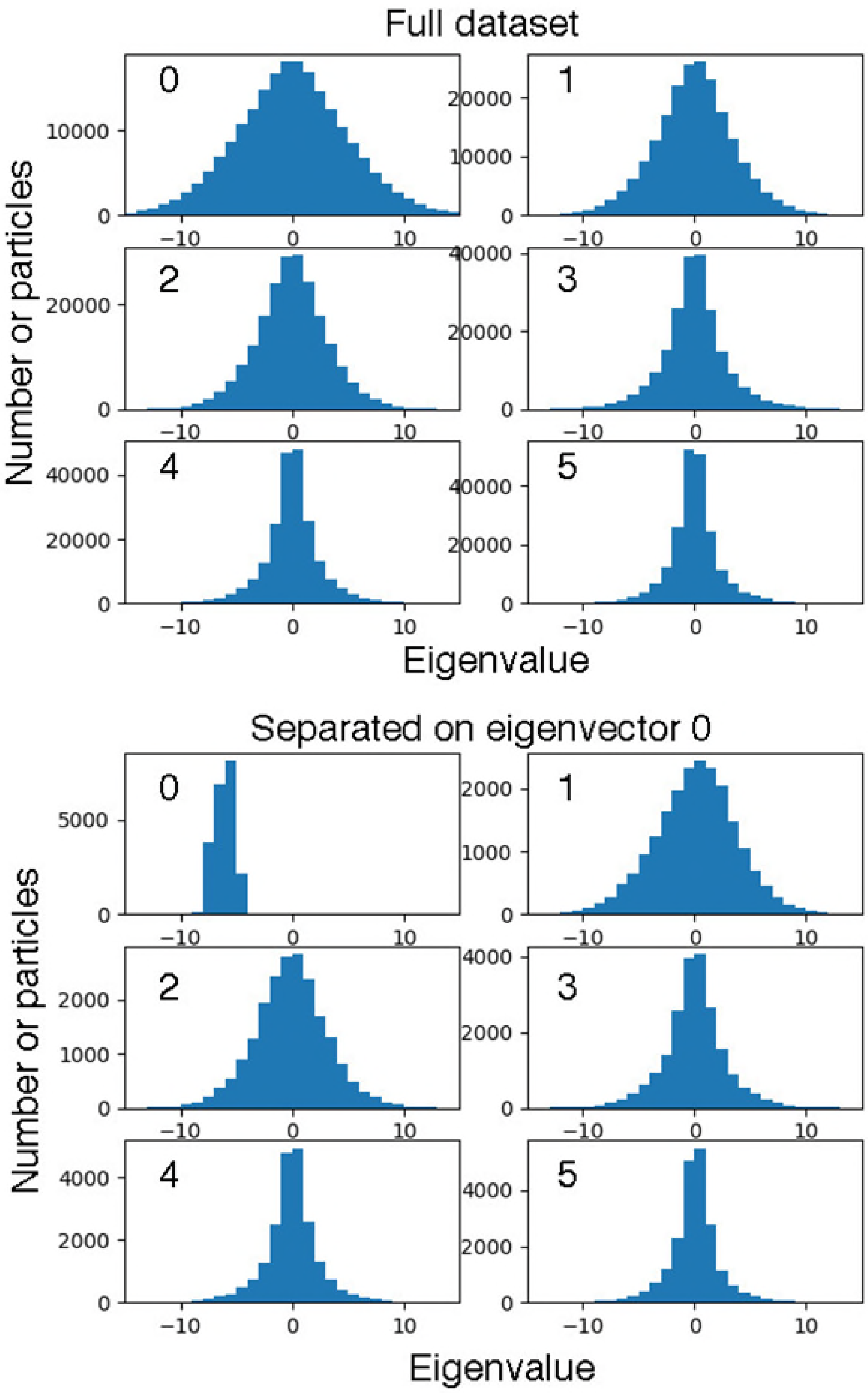

Supplementary Figure 8b. Eigenvalue distributions for class 0-2. Comparison of Eigenvalue distributions for the full dataset (top) and subclass 0-2 (bottom). Each subplot shows distributions of values for eigenvectors noted in the top left of the subplot.

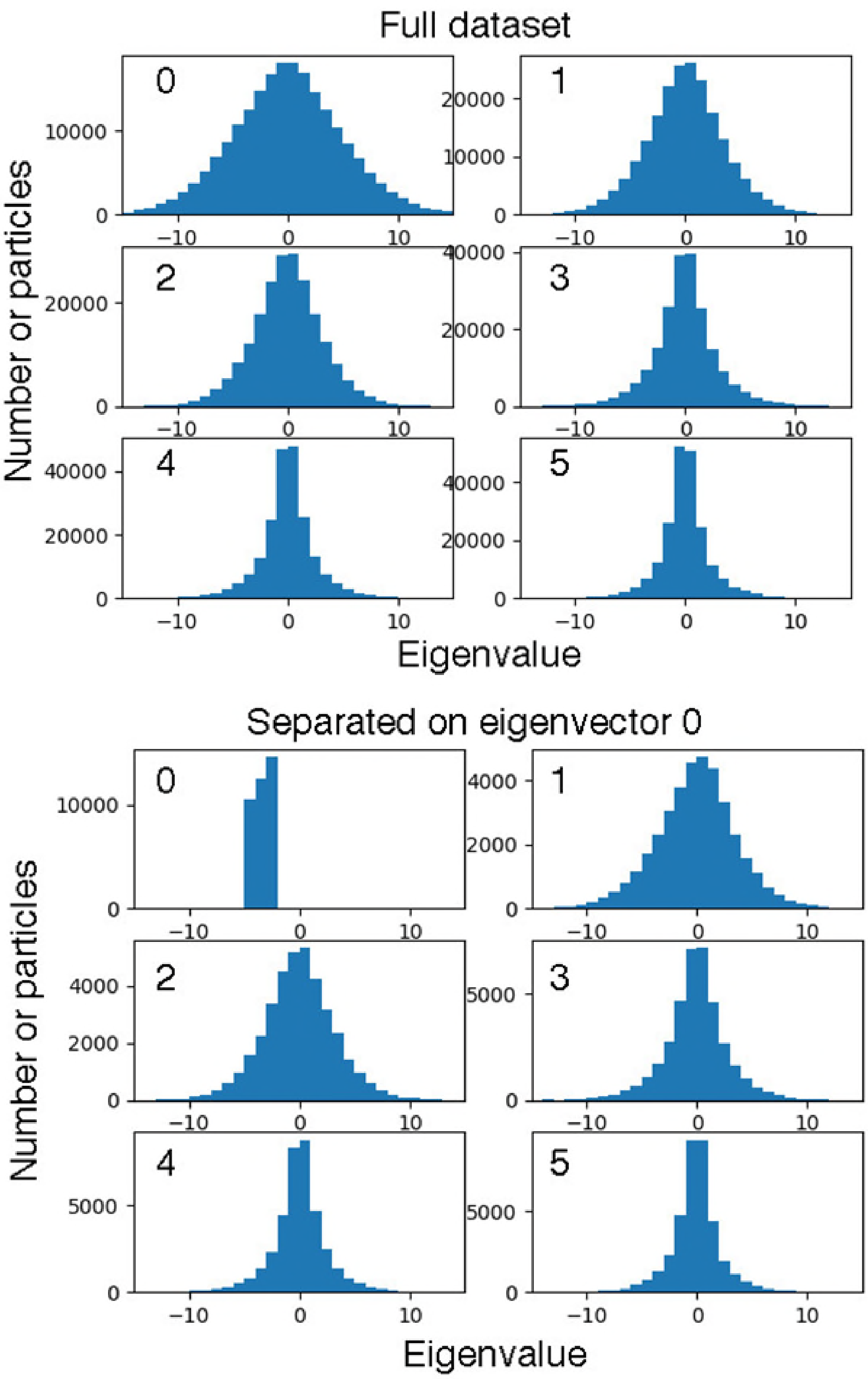

Supplementary Figure 8c. Eigenvalue distributions for class 0-3. Comparison of Eigenvalue distributions for the full dataset (top) and subclass 0-3 (bottom). Each subplot shows distributions of values for eigenvectors noted in the top left of the subplot.

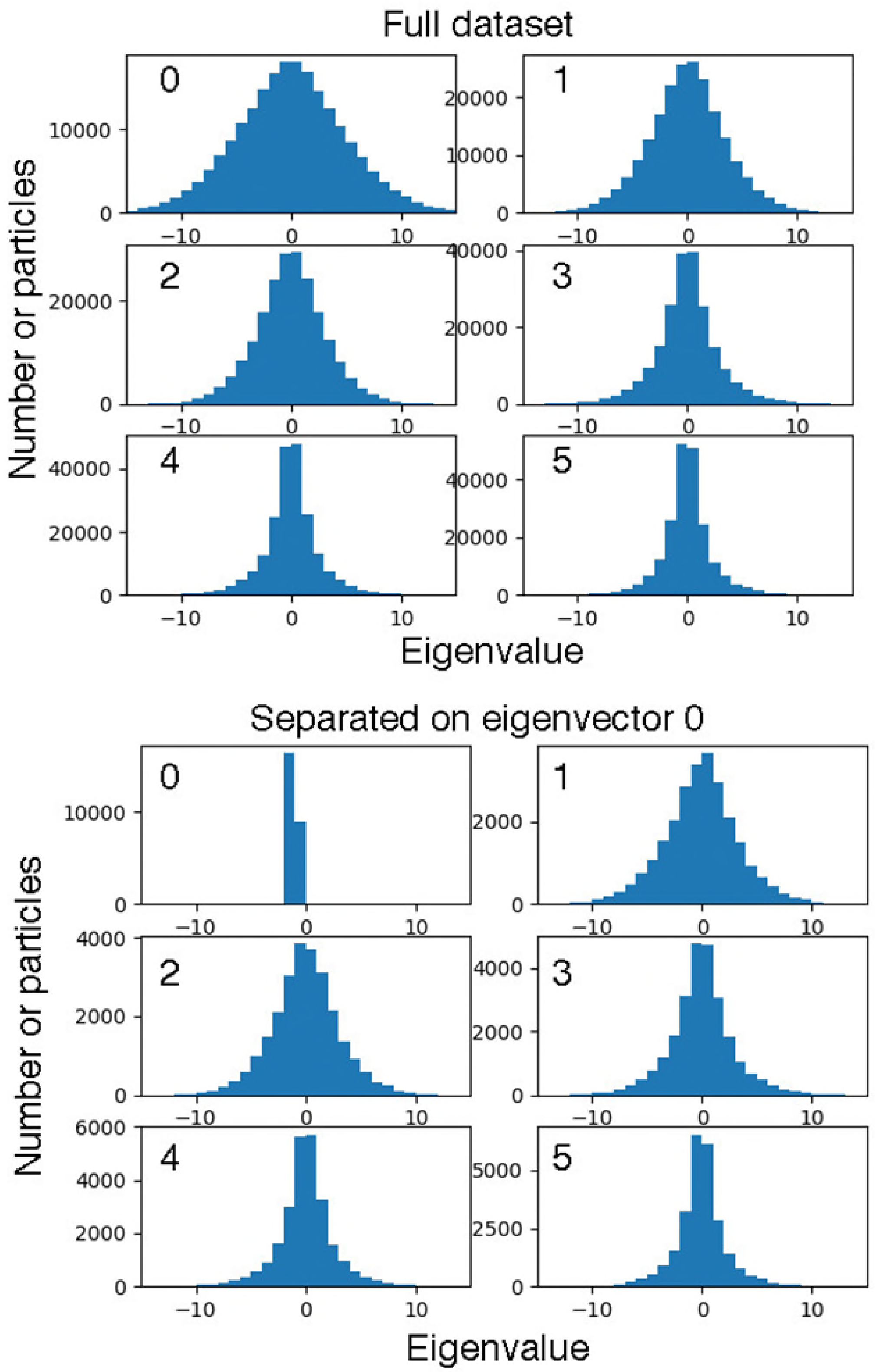

Supplementary Figure 8d. Eigenvalue distributions for class 0-4. Comparison of Eigenvalue distributions for the full dataset (top) and subclass 0-4 (bottom). Each subplot shows distributions of values for eigenvectors noted in the top left of the subplot.

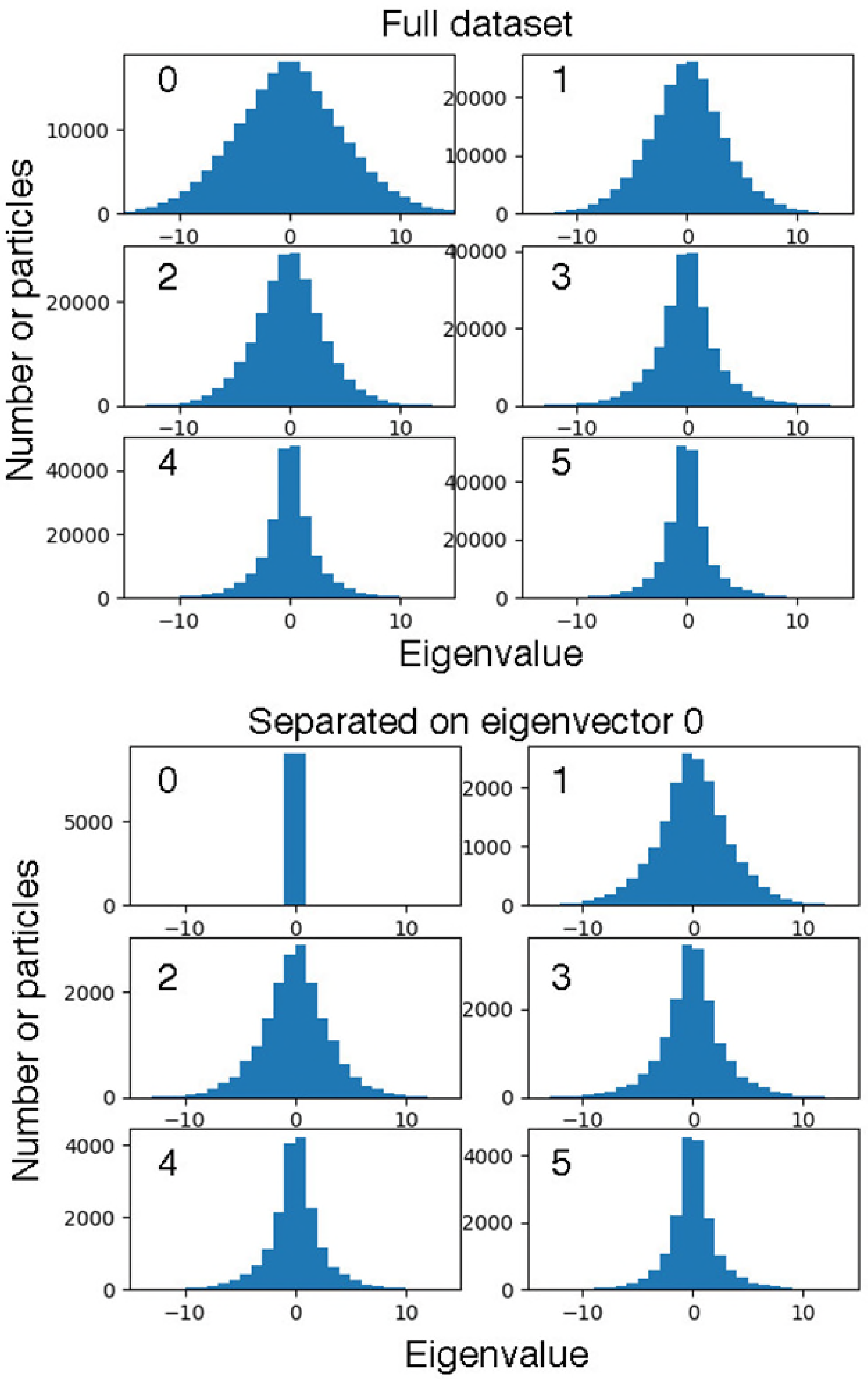

Supplementary Figure 8e. Eigenvalue distributions for class 0-5. Comparison of Eigenvalue distributions for the full dataset (top) and subclass 0-5 (bottom). Each subplot shows distributions of values for eigenvectors noted in the top left of the subplot.

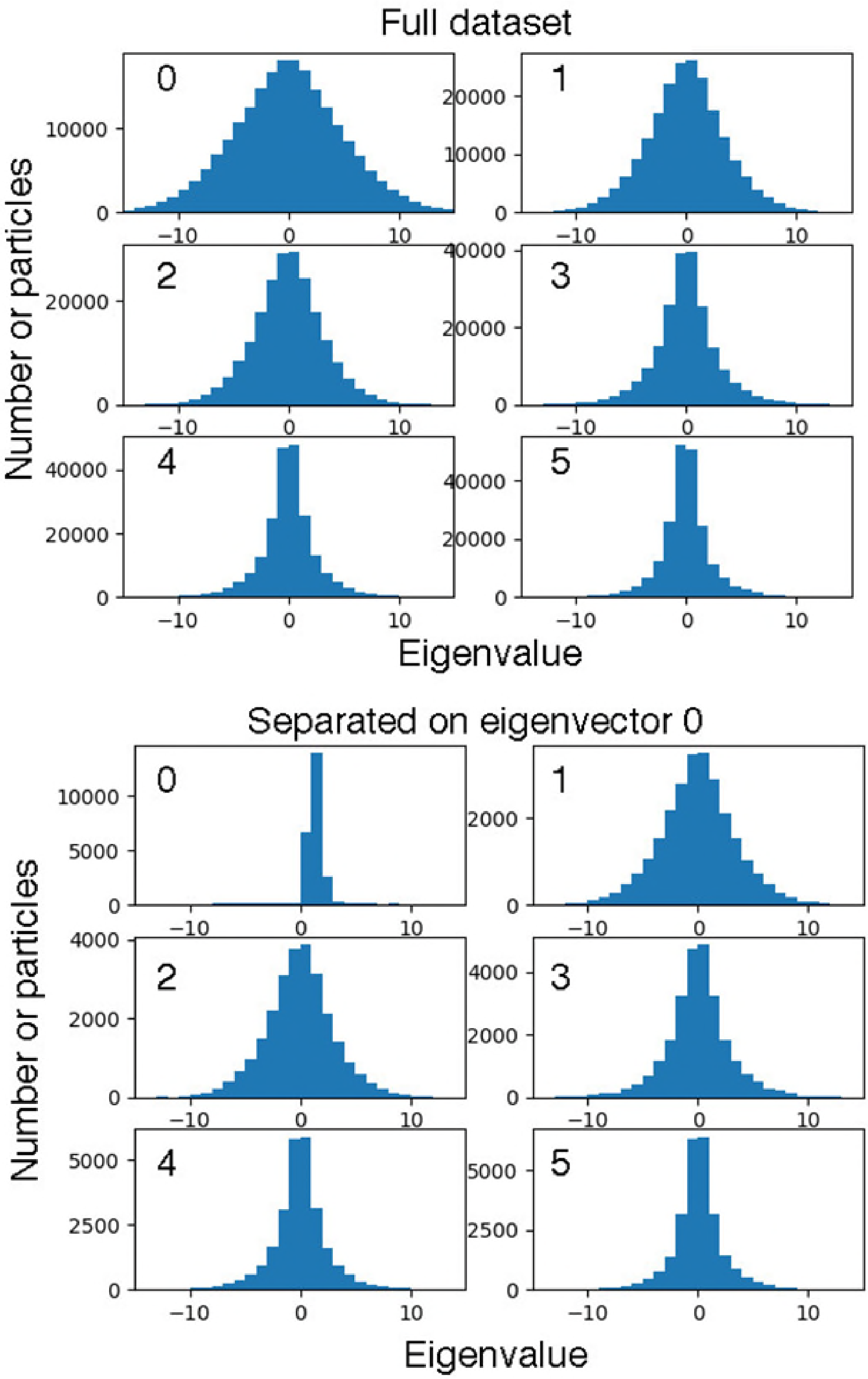

Supplementary Figure 8f. Eigenvalue distributions for class 0-6. Comparison of Eigenvalue distributions for the full dataset (top) and subclass 0-6 (bottom). Each subplot shows distributions of values for eigenvectors noted in the top left of the subplot.

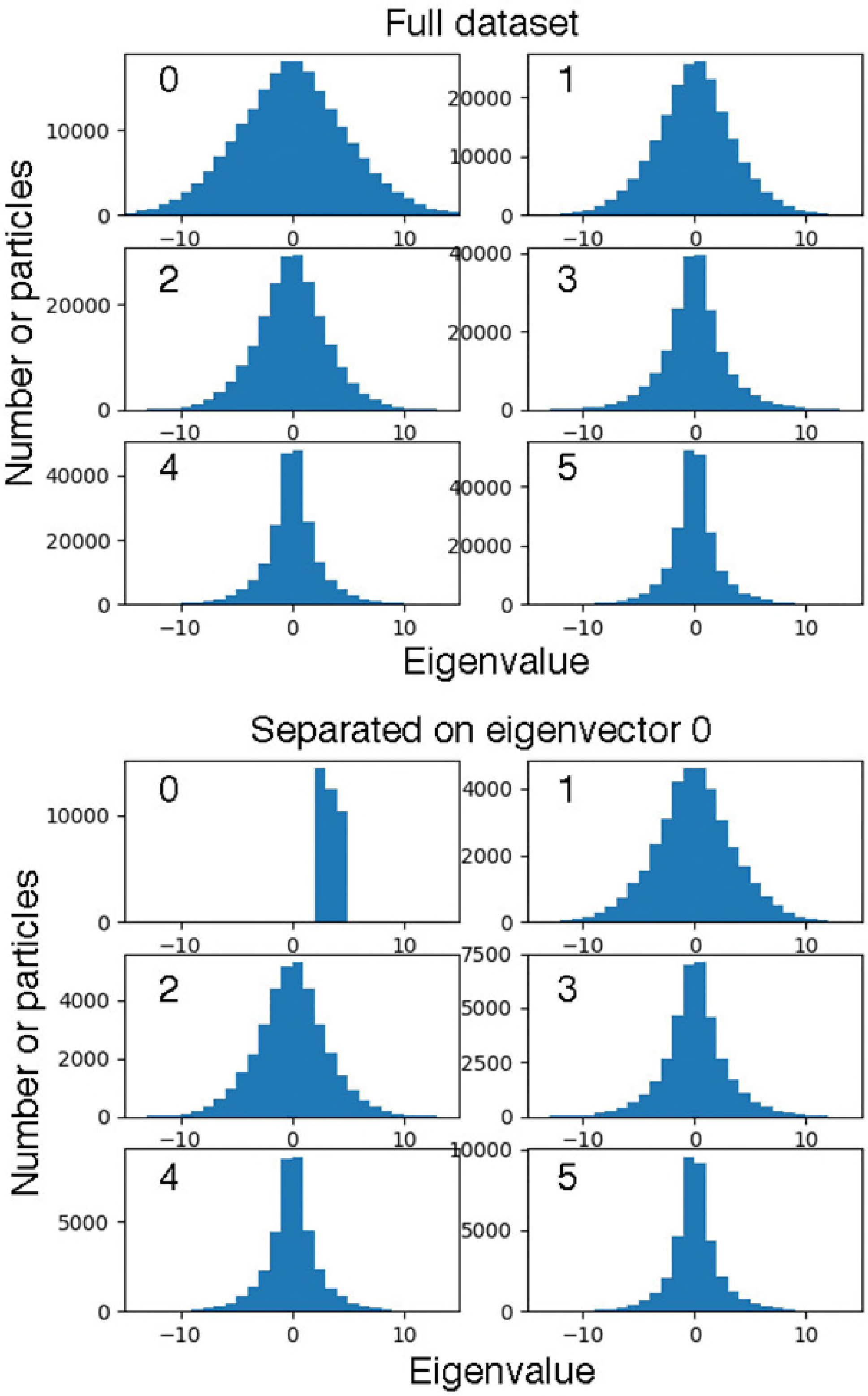

Supplementary Figure 8g. Eigenvalue distributions for class 0-7. Comparison of Eigenvalue distributions for the full dataset (top) and subclass 0-7 (bottom). Each subplot shows distributions of values for eigenvectors noted in the top left of the subplot.

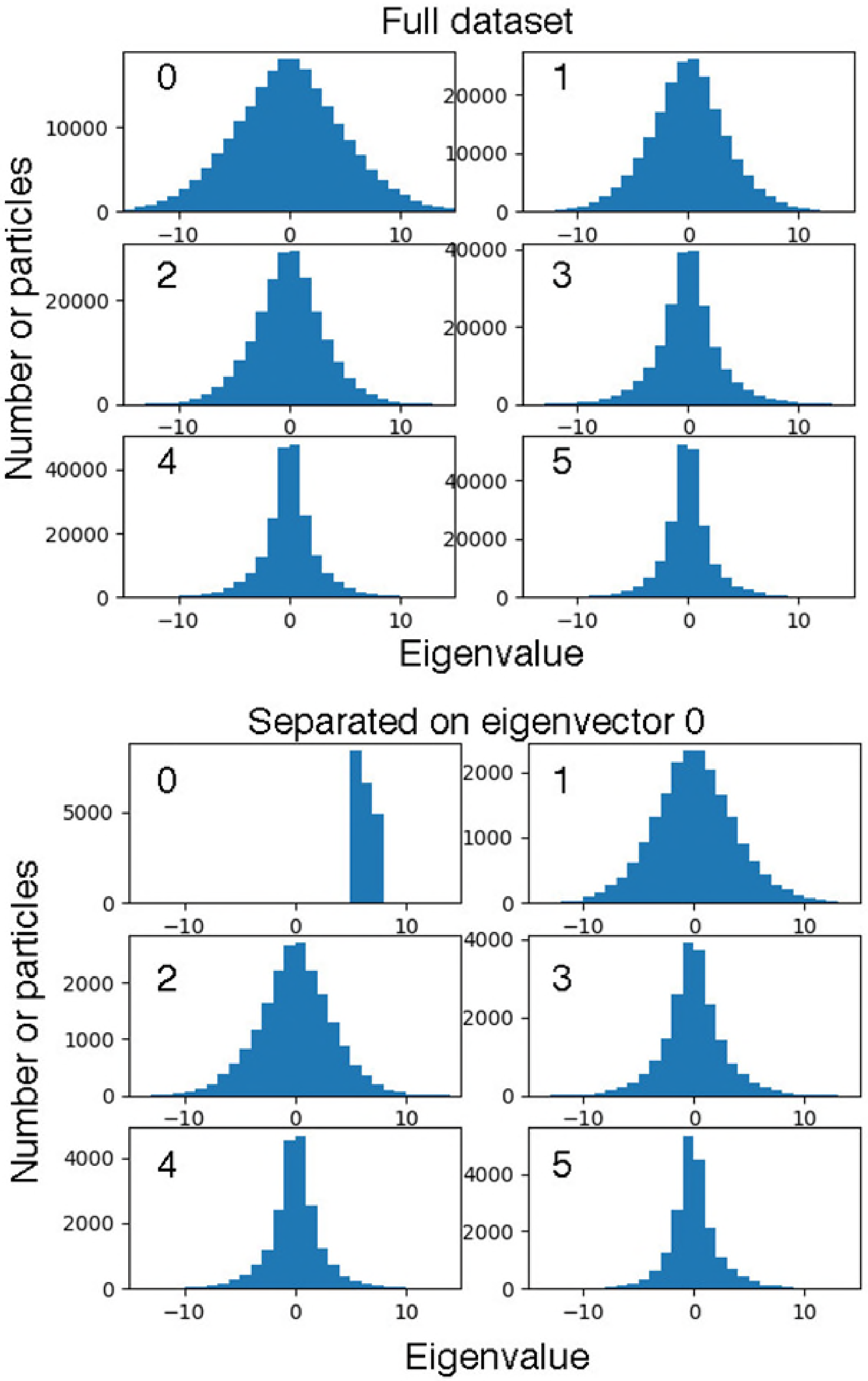

Supplementary Figure 8h. Eigenvalue distributions for class 0-8. Comparison of Eigenvalue distributions for the full dataset (top) and subclass 0-8 (bottom). Each subplot shows distributions of values for eigenvectors noted in the top left of the subplot.

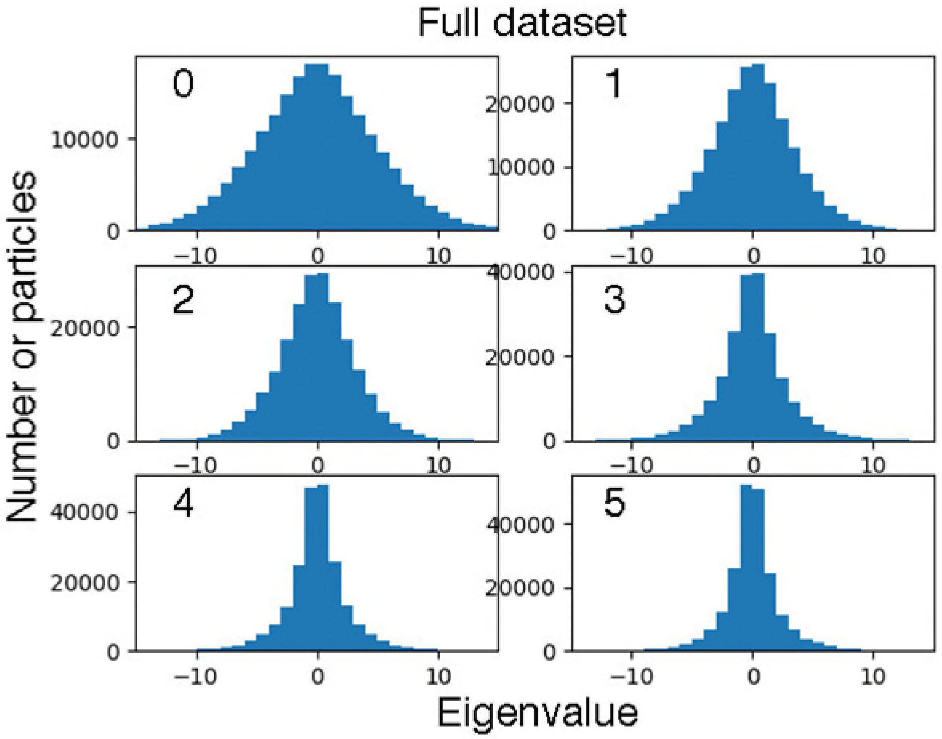

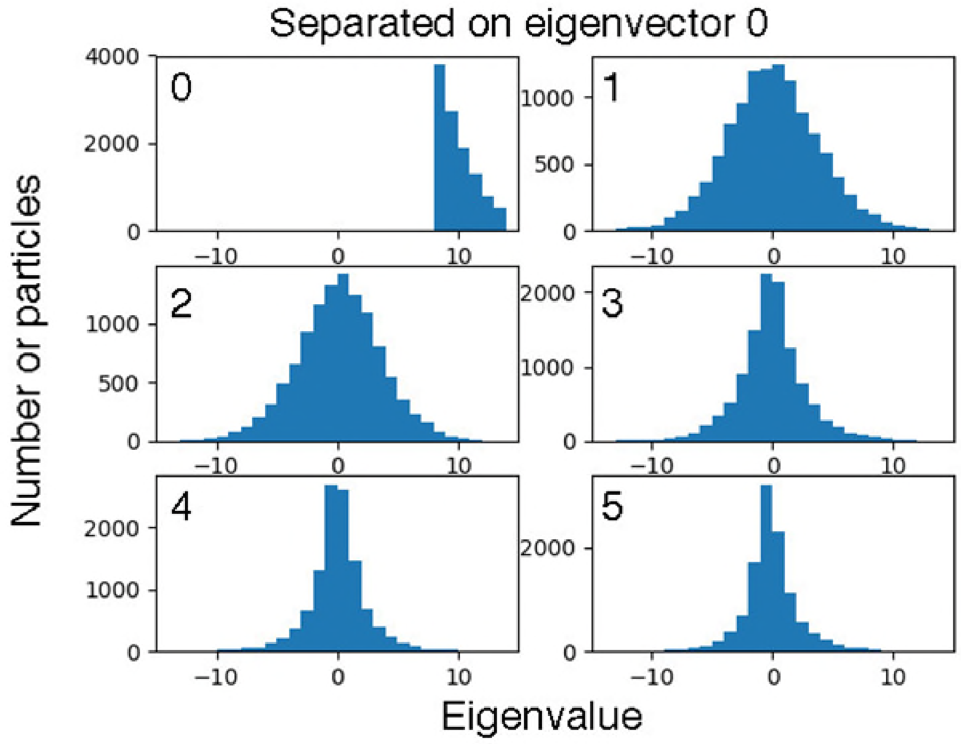

Supplementary Figure 8i. Eigenvalue distributions for class 0-9. Comparison of Eigenvalue distributions for the full dataset (top) and subclass 0-9 (bottom). Each subplot shows distributions of values for eigenvectors noted in the top left of the subplot.

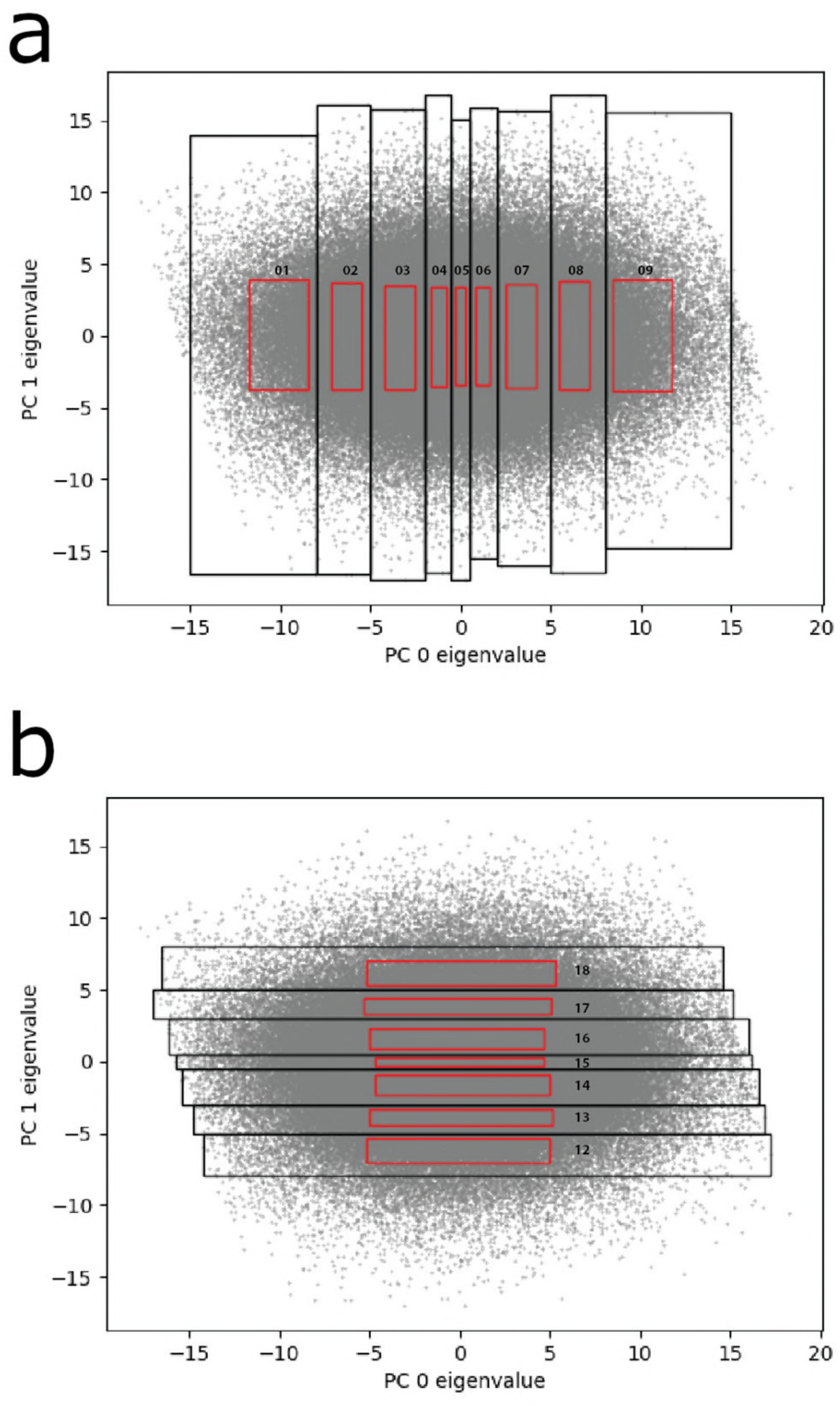

Supplementary Figure 9. Distributions of eigenvalues for the different classes. Grey clouds represent the distribution of eigenvalues for component 0 and component 1 in the entire dataset. For classes separated along (a) component 0 and (b) component 1 black boxes represent the full range of eigenvalues in each class and red boxes represent two standard deviations around the mean value.

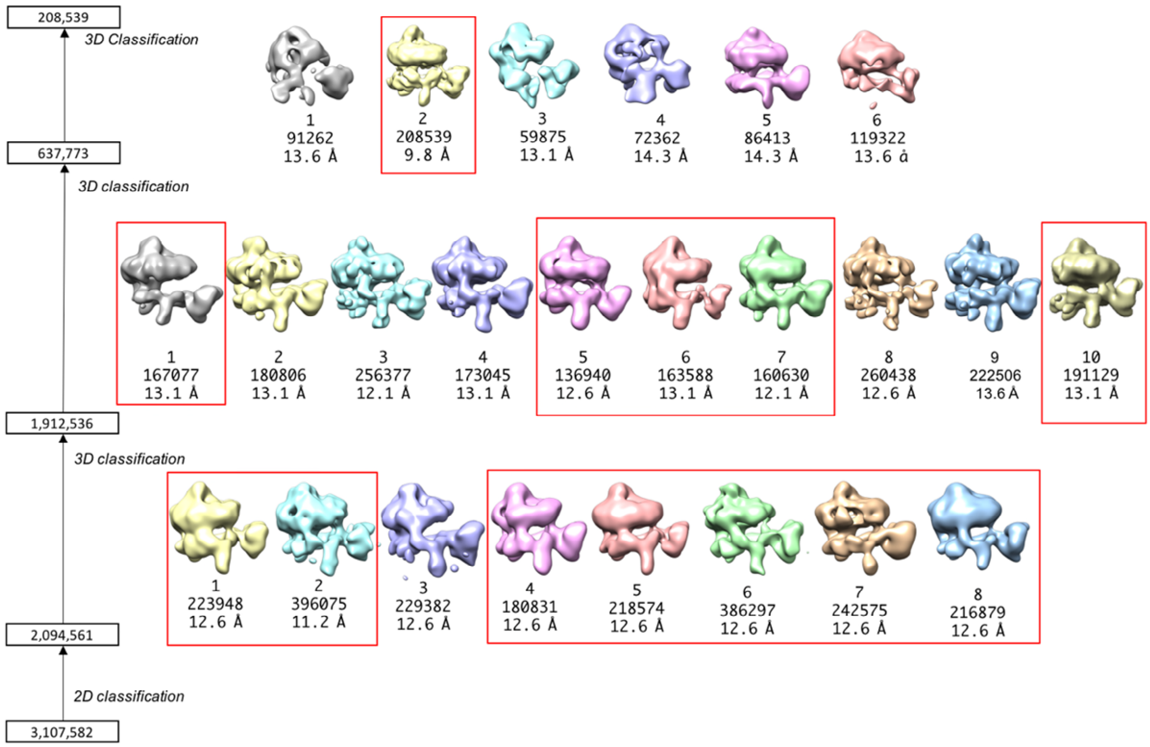

Supplementary Figure 10. Classification and selection of particles during reconstruction of the consensus map. Size of the data set at each step is listed on the left. Class number, number of particles and resolution are listed below each class. Classes taken forward to the next step are boxed in red.

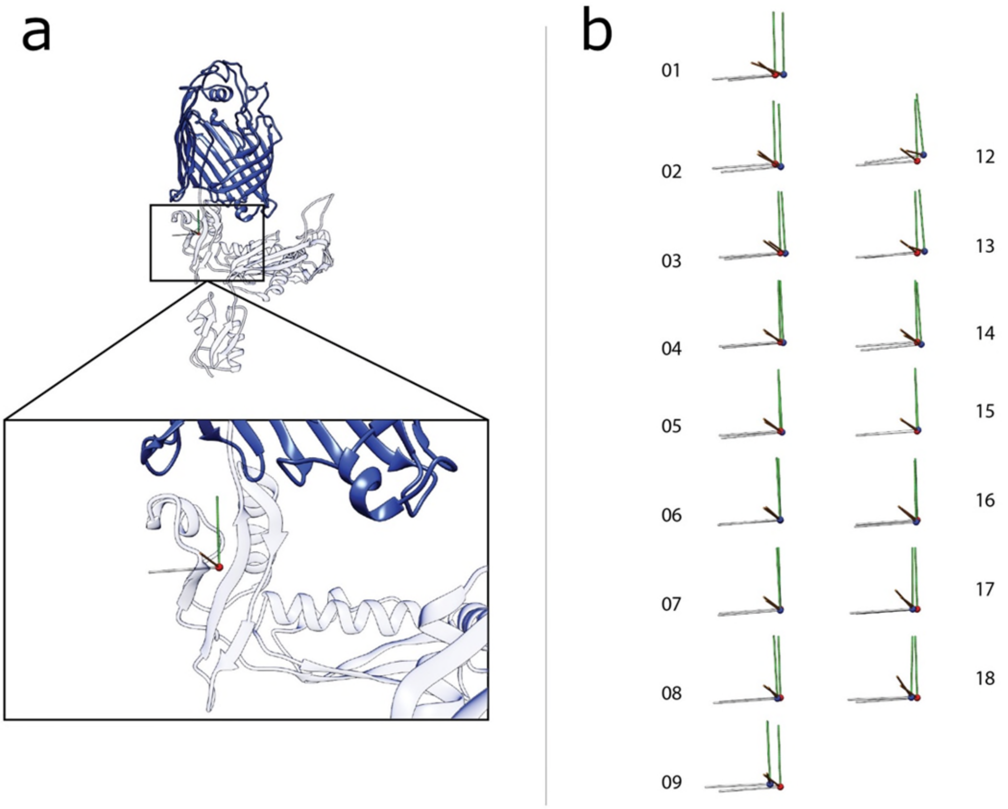

Supplementary Figure 11. Rotation and translation of POTRA 5 in nine different structures generated from separation along eigenvectors of component 0 and 1. (a) BamA from structure 0-9 overlaid on BamA from 5LJO[19]. Spheres mark the centres of mass of POTRA 5 from 5LJO[19] (red) and structure 0-9 (blue). Cylinders along arbitrary x, y, and z axes (white, green, and orange respectively) are drawn to aid in visualising the rotation between the two main eigenvalues. (b) Spheres and cylinders for all 16 structures as in panel (a). comparing the positions of POTRA 5 in 5LJO[19] (red) and the ensemble of structures.

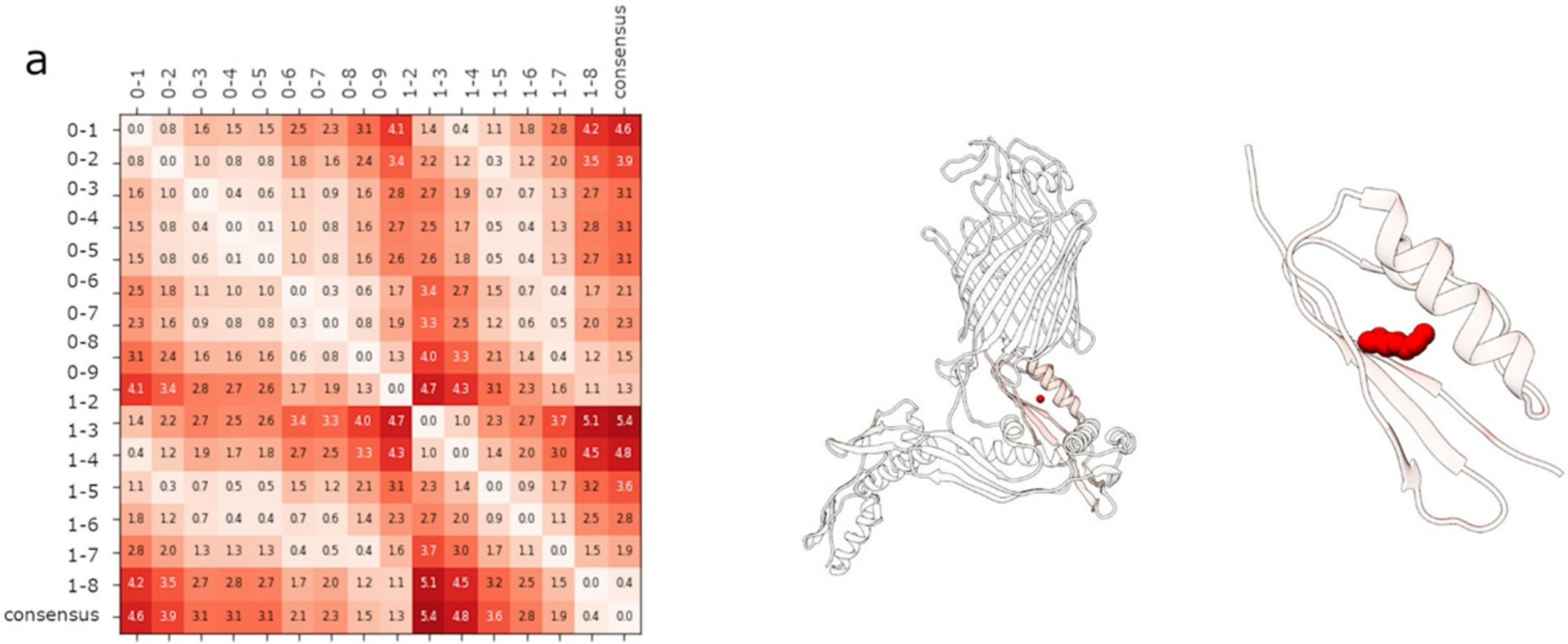

Supplementary Figure 12 Comparison of POTRA1-5 conformations. (a) Matrix showing the differences in centres of mass (in Å) of POTRA 5 for the 16 ensemble structures and consensus structure when aligned on the BamA β-barrel (left) Position of POTRA5 in the concensus structure with the centre of mass designated by a red sphere (centre) and overlay of the location of POTRA5 for all ensemble structures overlaid over the consensus structure, when aligned on the β-barrel.

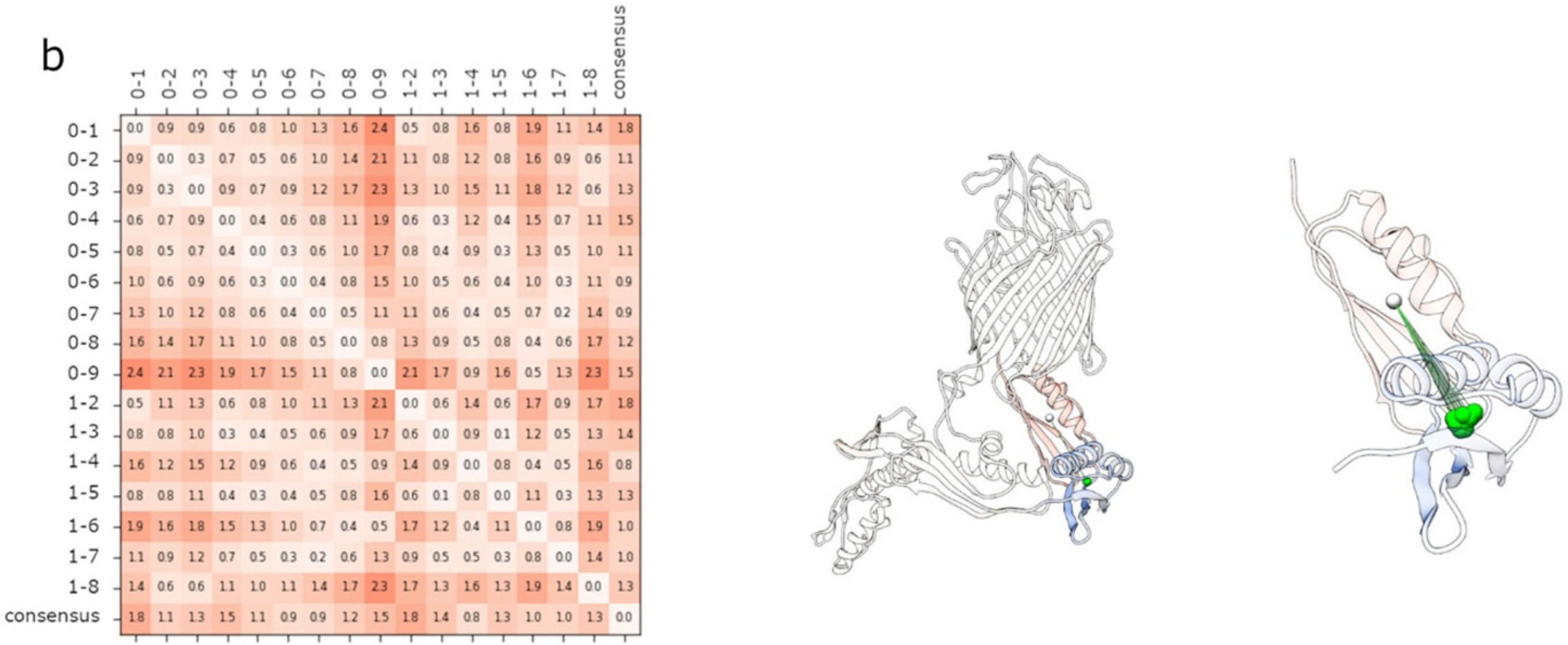

Supplementary Figure 12 (continued). Comparison of POTRA1-5 conformations. (b) Matrix showing the differences in centres of mass (in Å) of POTRA 4 for the 16 ensemble structures and consensus structure when aligned POTRA 5 (left) Positions of POTRAs 4 and 5 in the consensus structure with the centres of mass designated by green and white spheres respectively (centre) and overlay of the location of POTRAs 4 and 5 for all ensemble structures overlaid over the consensus structure, when aligned on POTRA 5.

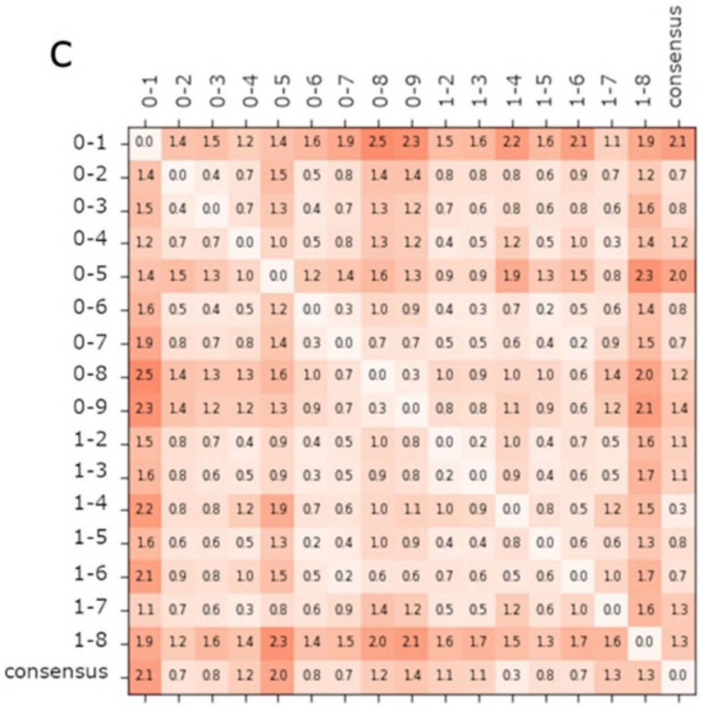

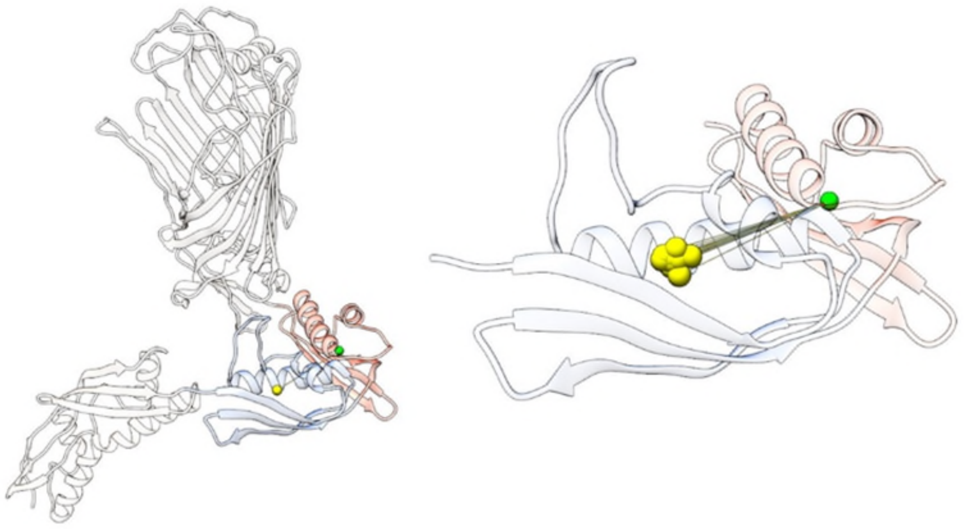

Supplementary Figure 12 (continued). Comparison of POTRA1-5 conformations. (c) Matrix showing the differences in centres of mass (in Å) of POTRA 3 for the 16 ensemble structures and consensus structure when aligned POTRA 4 (left) Positions of POTRAs 3 and 4 in the consensus structure with the centres of mass designated by yellow and green spheres respectively (centre) and overlay of the location of POTRAs 3 and 4 for all ensemble structures overlaid over the consensus structure, when aligned on POTRA 4.

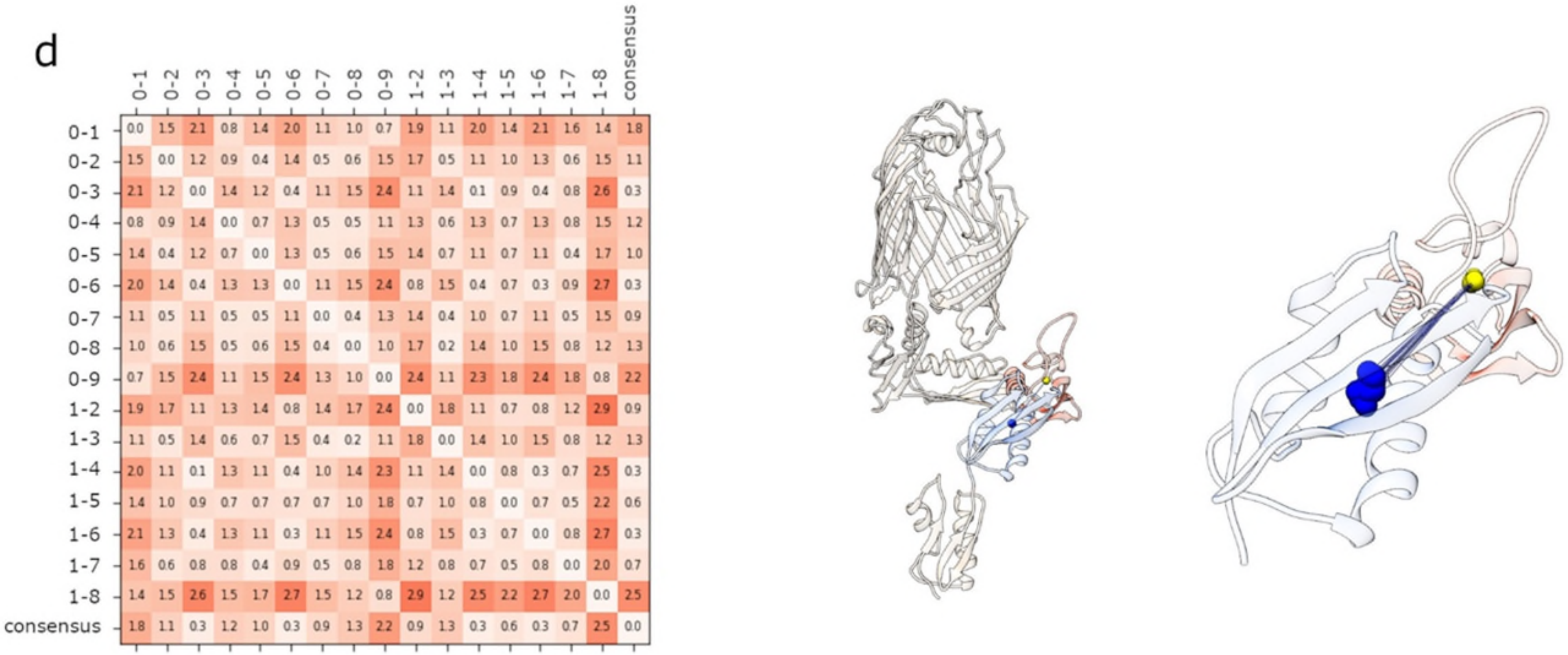

Supplementary Figure 12 (continued). Comparison of POTRA1-5 conformations. (d) Matrix showing the differences in centres of mass (in Å) of POTRA 2 for the 16 ensemble structures and consensus structure when aligned POTRA 3 (left) Positions of POTRAs 2 and 3 in the consensus structure with the centres of mass designated by blue and yellow spheres respectively (centre) and overlay of the location of POTRAs 2 and 3 for all ensemble structures overlaid over the consensus structure, when aligned on POTRA 3.

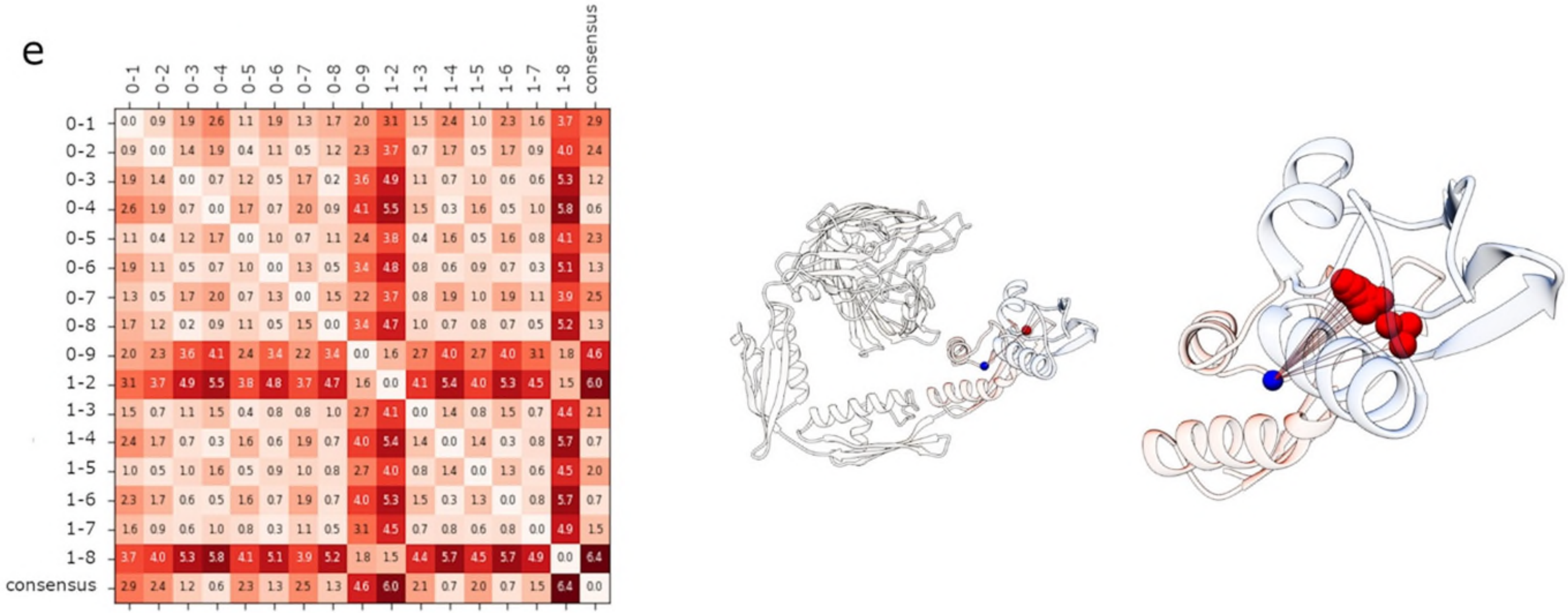

Supplementary Figure 12 (continued). Comparison of POTRA1-5 conformations. (e) Matrix showing the differences in centres of mass (in Å) of POTRA 1 for the 16 ensemble structures and consensus structure when aligned POTRA 2 (left) Positions of POTRAs 1 and 2 in the consensus structure with the centres of mass designated by red and blue spheres respectively (centre) and overlay of the location of POTRAs 1 and 2 for all ensemble structures overlaid over the consensus structure, when aligned on POTRA 2.

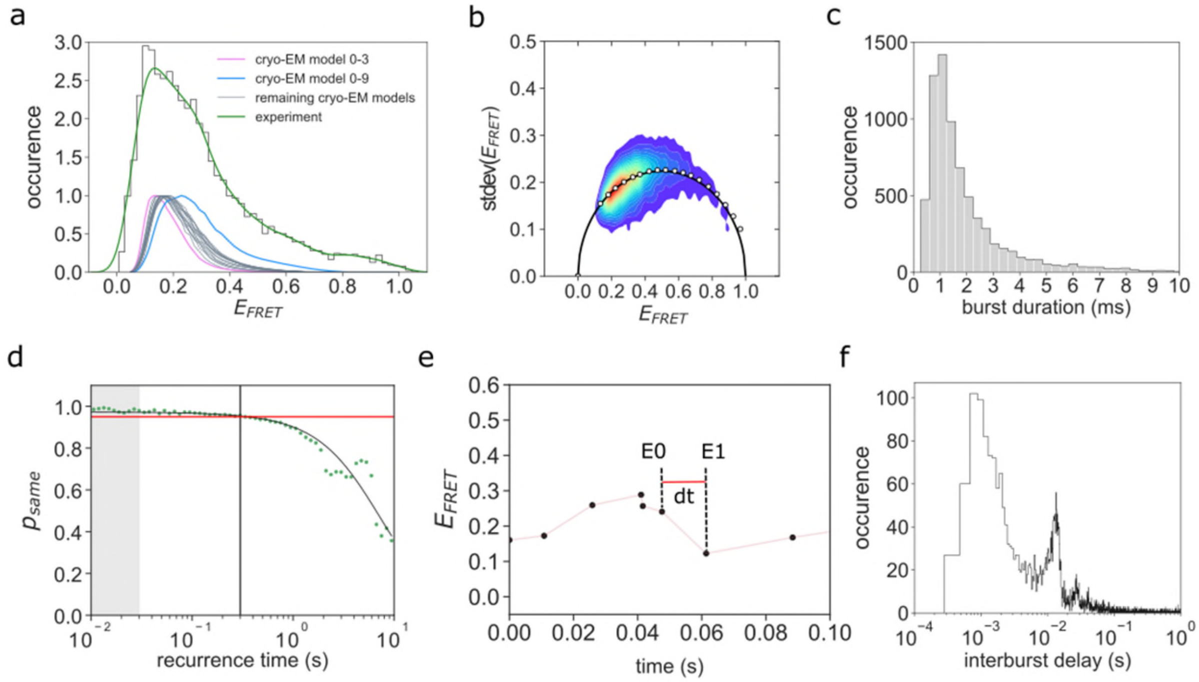

Supplementary Figure 13. Recurrence analysis of single particles. a) 1D histogram of all measured E_{FRET} values (experiment (green)) and predicted distributions for the 16 BAM nanodisc cryo-EM derived models and the BAM micelle cryo-EM derived model. b) We employed burst variance analysis (BVA) to look for dynamics on the timescale of diffusion - here ~1ms (see panel c). In BVA each burst is binned into aliquots of 5 photons and the E_{FRET} calculated for each bin, then plotted against the standard deviation of the sub-bursts in each bin. The black arc shows the standard deviation expected in E_{FRET} due to shot noise. There is no significant density above this line, which would indicate dynamic interchange of states on a sub-millisecond timescale (note also that the standard deviation within each burst is low). d) At the low BAM concentration used here ~20 pM (and substantially less after post acquisition removal of all of the singly labelled bursts as described in the Methods), the recurrence of an individual molecule is more likely (recurrence probability, P_{same}>0.95 horizontal red line) to be observed within a short time of the first passage of that molecule than the arrival of a new molecule. e) Thus, for short temporal windows (here calculated as up to 300 ms vertical grey line in (d)), any two consecutive bursts ‘E0’ and ‘E1’ with very different E_{FRET} values represent instances of conformational change occurring on the

timescale of the inter-burst delay (dt, red horizontal line). f) A histogram of all of the inter-burst delays that occurred reveals this timescale to be ~ 1-30 ms (largest two populations), while the second peak likely reflects the upper limit of time between bursts after which diffusion away from the confocal volume becomes far more likely than recurrence. Pairs identified in this manner with inter-burst delays up to 30 ms (grey shaded area in (d)) are presented in the main text, Figure 6 b) fitted to a 2D Kernel Density Estimate (KDE) using the kdeplot function implemented in the python library 'seaborn' for python 3.7 as described in the methods section.

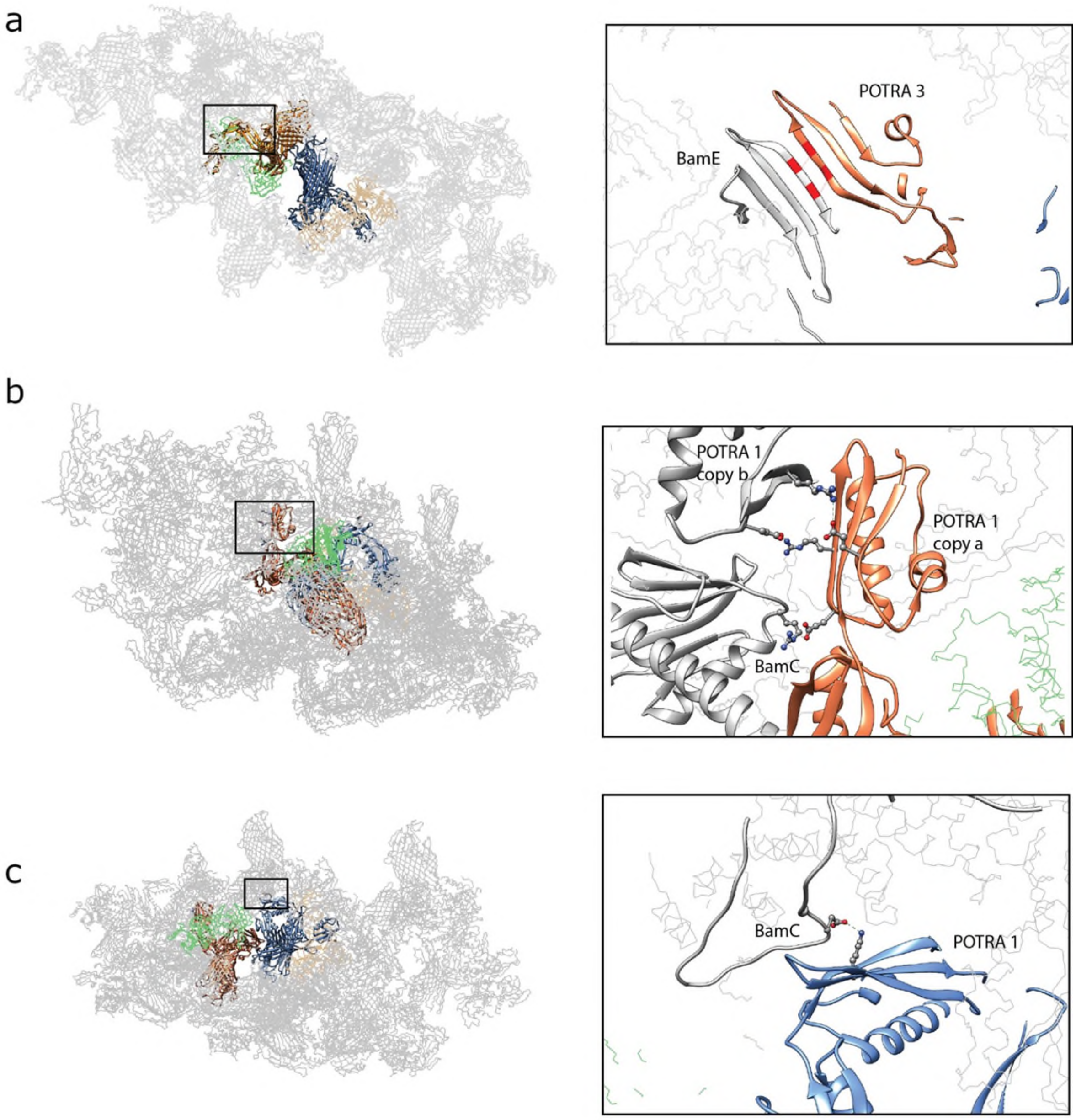

Supplementary Figure 14. Different crystal packing interactions affecting the POTRA conformations in BamACDE crystal structures. The two copies (‘a’ (orange) and ‘b’ (blue)) in the unit cell of 5D0Q[21] and 5EKQ[22] (a) β-augumentation by POTRA 3 in copy ‘a’ of 5D0Q[21], which is not present in copy ‘b’. (b) A salt bridge between POTRA1 of the two copies in the unit cell of 5D0Q[21] and (c) a salt bridge between POTRA 1 and BamC in 5EKQ[22].

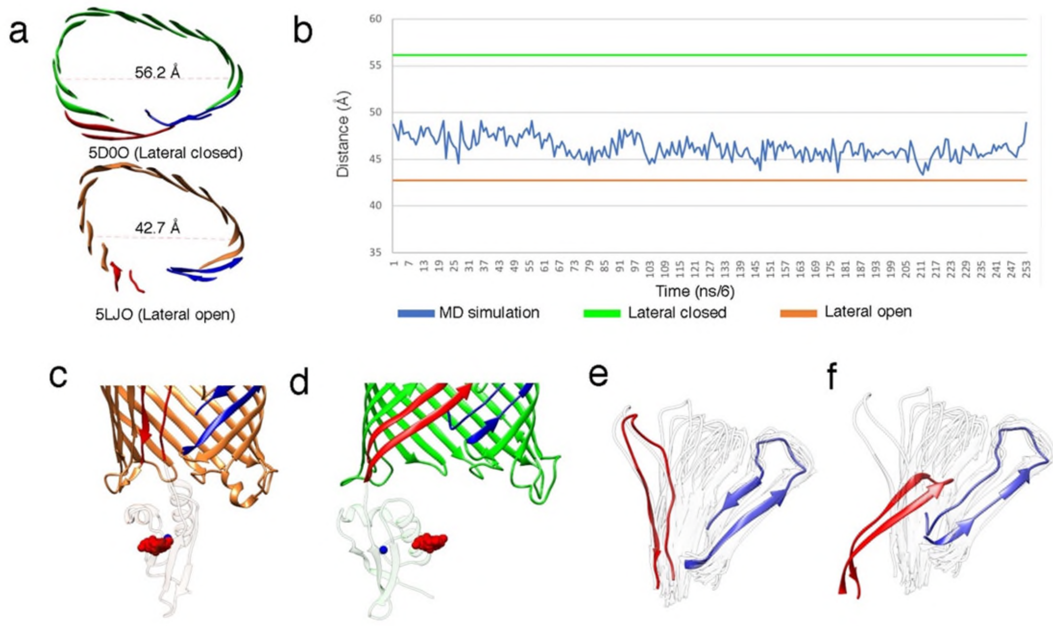

Supplementary Figure 15. Analysis of the conformation of the BAM complex over the course of the MD simulation. (a) Illustration of the shape of the BamA β-barrel in the lateral closed (top) and lateral open (bottom) conformations with the distance between residues S_{432} and F_{737} noted in each. (b) The S_{432} to F_{737} distance over the course of the simulation (blue), compared to the reference distances for lateral open (orange) and lateral closed (green). Location of POTRA5 in the (c) lateral open (EM structure 5LJO) and (d) lateral closed (X-ray structure 5D0O) conformations. Blue spheres represent the centres of mass for POTRA5 in the EM and crystal structures, respectively; red spheres represent the centres of mass of POTRA5 sampled over the simulation as in panel (b). Conformation of the lateral gate (β-strands 1/2 - red and 15/16 - blue) in the (e) lateral open and (f) lateral closed conformations with the BamA β-barrel aligned on the invariant back half (residues 518-745). Grey transparent models represent the lateral gate structure sampled over the course of the simulation as in panel (b).

Supplementary References

1 Gao, Y., Cao, E., Julius, D. & Cheng, Y. TRPV1 structures in nanodiscs reveal mechanisms of ligand and lipid action. *Nature* **534**, 347-351, doi:10.1038/nature17964 (2016).

2 Shen, P. S. *et al.* The Structure of the Polycystic Kidney Disease Channel PKD2 in Lipid Nanodiscs. *Cell* **167**, 763-773 e711, doi:10.1016/j.cell.2016.09.048 (2016).

3 Willegems, K. & Efremov, R. G. Influence of Lipid Mimetics on Gating of Ryanodine Receptor. *Structure* **26**, 1303-1313 e1304, doi:10.1016/j.str.2018.06.010 (2018).

4 Matthies, D. *et al.* Single-particle cryo-EM structure of a voltage-activated potassium channel in lipid nanodiscs. *Elife* **7**, e37558, doi:10.7554/eLife.37558 (2018).

5 Zhao, D. Y. *et al.* Cryo-EM structure of the native rhodopsin dimer in nanodiscs. *J Biol Chem* **294**, 14215-14230, doi:10.1074/jbc.RA119.010089 (2019).

6 Laverty, D. *et al.* Cryo-EM structure of the human alpha1beta3gamma2 GABAA receptor in a lipid bilayer. *Nature* **565**, 516-520, doi:10.1038/s41586-018-0833-4 (2019).

7 Burendei, B. *et al.* Cryo-EM structures of undocked innexin-6 hemichannels in phospholipids. *Sci. Adv.* **6**, eaax3157, doi:10.1126/sciadv.aax3157 (2020).

8 Jackson, S. M. *et al.* Structural basis of small-molecule inhibition of human multidrug transporter ABCG2. *Nat. Struct. Mol. Biol.* **25**, 333-340, doi:10.1038/s41594-018-0049-1 (2018).

9 Efremov, R. G., Leitner, A., Aebersold, R. & Raunser, S. Architecture and conformational switch mechanism of the ryanodine receptor. *Nature* **517**, 39-43, doi:10.1038/nature13916 (2015).

10 Frauenfeld, J. *et al.* Cryo-EM structure of the ribosome-SecYE complex in the membrane environment. *Nat. Struct. Mol. Biol.* **18**, 614-621, doi:10.1038/nsmb.2026 (2011).

11 Chen, Q. *et al.* Structure of mammalian endolysosomal TRPML1 channel in nanodiscs. *Nature* **550**, 415-418, doi:10.1038/nature24035 (2017).

12 Jin, P. *et al.* Electron cryo-microscopy structure of the mechanotransduction channel NOMPC. *Nature* **547**, 118-122, doi:10.1038/nature22981 (2017).

13 McGoldrick, L. L. *et al.* Opening of the human epithelial calcium channel TRPV6. *Nature* **553**, 233-237, doi:10.1038/nature25182 (2018).

14 Feng, S. *et al.* Cryo-EM Studies of TMEM16F Calcium-Activated Ion Channel Suggest Features Important for Lipid Scrambling. *Cell Rep* **28**, 567-579 e564, doi:10.1016/j.celrep.2019.06.023 (2019).

15 Flores, J. A. *et al.* Connexin-46/50 in a dynamic lipid environment resolved by CryoEM at 1.9 Å. *bioRxiv*, doi:10.1101/2020.04.14.036384 (2020).

16 Kalienkova, V. *et al.* Stepwise activation mechanism of the scramblase nhTMEM16 revealed by cryo-EM. *Elife* **8**, e44364, doi:10.7554/eLife.44364 (2019).

17 Kathryn A. Diederichs, X. N., Sarah E. Rollauer, Istvan Botos, Xiaofeng Tan, Martin S. King, Edmund R.S. Kunji, Jiansen Jiang, Susan K. Buchanan. Structural Insight into Mitochondrial β-Barrel Outer Membrane Protein Biogenesis. *bioRxiv* **2020.04.09.034678**, doi:10.1101/2020.04.09.034678 (2020).

18 Denisov, I. G., Grinkova, Y. V., Lazarides, A. A. & Sligar, S. G. Directed self-assembly of monodisperse phospholipid bilayer nanodiscs with controlled size. *J. Am. Chem. Soc.* **126**, 3477-3487, doi: 10.1021/ja0393574 (2004).

19 Iadanza, M. G. *et al.* Lateral opening in the intact beta-barrel assembly machinery captured by cryo-EM. *Nat. Commun.* **7**, 12865, doi:10.1038/ncomms12865 (2016).
20 Pautsch, A. & Schulz, G. E. High-resolution structure of the OmpA membrane domain. *J. Mol. Bio.* **298**, 273-282, doi:10.1006/jmbi.2000.3671 (2000).
21 Gu, Y. *et al.* Structural basis of outer membrane protein insertion by the BAM complex. *Nature* **531**, 64-69, doi:10.1038/nature17199 (2016).
22 Bakelar, J., Buchanan, S. K. & Noinaj, N. The structure of the β-barrel assembly machinery complex. *Science* **351**, 180-186, doi:10.1126/science.aad3460 (2016).
