# Supplementary material for: Distortion of the bilayer and dynamics of the BAM complex in lipid nanodiscs
Source: Commun Biol. 2020 Dec 14;3:766. doi: 10.1038/s42003-020-01419-w (PMC7736308; doi:10.1038/s42003-020-01419-w)
Supplement: Supplementary file 2 — Description of Additional Supplementary Files [file 42003_2020_1419_MOESM2_ESM.pdf]

Description of Additional Supplementary Files

File Name: Supplementary Movie 1
Description: Principal component 0. Animation showing the two refined bodies from the multibody refinement transformed by Eigenvectors described by principal component 0. The 10 frames represent evenly spaced intervals between the two extremes of the vectors.

File Name: Supplementary Movie 2
Description: Principal component 1 Animation showing the two refined bodies from the multibody refinement transformed by Eigenvectors described by principal component 1. The 10 frames represent evenly spaced intervals between the two extremes of the vectors.

File Name: Supplementary Movie 3
Description: Principal component 2 Animation showing the two refined bodies from the multibody refinement transformed by Eigenvectors described by principal component 2. The 10 frames represent evenly spaced intervals between the two extremes of the vectors.

File Name: Supplementary Movie 4
Description: Principal component 3 Animation showing the two refined bodies from the multibody refinement transformed by Eigenvectors described by principal component 3. The 10 frames represent evenly spaced intervals between the two extremes of the vectors.

File Name: Supplementary Movie 5
Description: Principal component 4 Animation showing the two refined bodies from the multibody refinement transformed by Eigenvectors described by principal component 4. The 10 frames represent evenly spaced intervals between the two extremes of the vectors.

File Name: Supplementary Movie 6
Description: Principal component 5 Animation showing the two refined bodies from the multibody refinement transformed by Eigenvectors described by principal component 5. The 10 frames represent evenly spaced intervals between the two extremes of the vectors.
