# Supplementary material for: Distortion of the bilayer and dynamics of the BAM complex in lipid nanodiscs
Source: Commun Biol. 2020 Dec 14;3:766. doi: 10.1038/s42003-020-01419-w (PMC7736308; doi:10.1038/s42003-020-01419-w)
Supplement: Supplementary file 9 — Reporting Summary [file 42003_2020_1419_MOESM9_ESM.pdf]

nature research

Corresponding author(s): Sheena E Radford
Neil A Ranson

Last updated by author(s): Jul 30, 2020

# Reporting Summary

Nature Research wishes to improve the reproducibility of the work that we publish. This form provides structure for consistency and transparency in reporting. For further information on Nature Research policies, see our Editorial Policies and the Editorial Policy Checklist.

## Statistics

For all statistical analyses, confirm that the following items are present in the figure legend, table legend, main text, or Methods section.

| n/a | Confirmed | |
|---|---|---|
| [x] | [ ] | The exact sample size (*n*) for each experimental group/condition, given as a discrete number and unit of measurement |
| [x] | [ ] | A statement on whether measurements were taken from distinct samples or whether the same sample was measured repeatedly |
| [x] | [ ] | The statistical test(s) used AND whether they are one- or two-sided<br>*Only common tests should be described solely by name; describe more complex techniques in the Methods section.* |
| [x] | [ ] | A description of all covariates tested |
| [x] | [ ] | A description of any assumptions or corrections, such as tests of normality and adjustment for multiple comparisons |
| [x] | [ ] | A full description of the statistical parameters including central tendency (e.g. means) or other basic estimates (e.g. regression coefficient) AND variation (e.g. standard deviation) or associated estimates of uncertainty (e.g. confidence intervals) |
| [x] | [ ] | For null hypothesis testing, the test statistic (e.g. *F*, *t*, *r*) with confidence intervals, effect sizes, degrees of freedom and *P* value noted<br>*Give P values as exact values whenever suitable.* |
| [x] | [ ] | For Bayesian analysis, information on the choice of priors and Markov chain Monte Carlo settings |
| [x] | [ ] | For hierarchical and complex designs, identification of the appropriate level for tests and full reporting of outcomes |
| [x] | [ ] | Estimates of effect sizes (e.g. Cohen's *d*, Pearson's *r*), indicating how they were calculated |

*Our web collection on statistics for biologists contains articles on many of the points above.*

## Software and code

Policy information about availability of computer code

Data collection

For CryoEM data collection:
ThermoFisher:
EPU 1.8
TEM software (2.9.1)
TEM Imaging Analysis (TIA)(4.17)
FluCam (6.9.1)
AutoCTF (0.6.9)

GATAN:
GMS 3/Digital Micrograph (DM)(3.22)

Data analysis

For CryoEM data analysis and structure refinement:
RELION v2.1
RELION v3.0
Motioncorr v2
gCTF v1.06
Phenix v 1.17.1
Coot v0.9-pre
Molprobity v4.5

For manuscripts utilizing custom algorithms or software that are central to the research but not yet described in published literature, software must be made available to editors and reviewers. We strongly encourage code deposition in a community repository (e.g. GitHub). See the Nature Research guidelines for submitting code & software for further information.

## Data

Policy information about availability of data

All manuscripts must include a data availability statement. This statement should provide the following information, where applicable:

- Accession codes, unique identifiers, or web links for publicly available datasets
- A list of figures that have associated raw data
- A description of any restrictions on data availability

Full data availability statement included:

Data Availability
The cryoEM map and fitted model for the consensus structure are available from the PDB and EMDB accession numbers 6SMX and EMD-10247. The 16 maps and models 0-1 through 1-8 from the ensemble analysis are available as PDB entries 6SNO, 6SN2, 6SN3, 6SN4, 6SN5, 6SN7, 6SN8, 6SN9, 6SOA, 6SO7, 6SO8, 6SOB, 6SOC, 6SOG, 6SOH, and 2SOJ and EMDB entries 10248, 10249, 10250, 10251, 10252, 10253, 10254, 10255, 10270, 10268, 10269, 10271, 10272, 10274, 10275, and 10276, respectively. The original 3107582 autopicked particles from which the dataset for this paper were drawn are available via the EMPIAR database (Deposition code 680).

# Field-specific reporting

Please select the one below that is the best fit for your research. If you are not sure, read the appropriate sections before making your selection.

[x] Life sciences [ ] Behavioural & social sciences [ ] Ecological, evolutionary & environmental sciences

For a reference copy of the document with all sections, see nature.com/documents/nr-reporting-summary-flat.pdf

# Life sciences study design

All studies must disclose on these points even when the disclosure is negative.

| | |
|---|---|
| Sample size | No statistical methods were used to predetermine sample size. |
| Data exclusions | No data exclusions were needed |
| Replication | The replicates of data gave similar results. |
| Randomization | This is not relevant to our study, because no grouping was needed. |
| Blinding | Investigators were not blinded to group allocation, as no grouping was needed for this study. |

# Reporting for specific materials, systems and methods

We require information from authors about some types of materials, experimental systems and methods used in many studies. Here, indicate whether each material, system or method listed is relevant to your study. If you are not sure if a list item applies to your research, read the appropriate section before selecting a response.

## Materials & experimental systems

| n/a | Involved in the study |
|---|---|
| ☒ | ☐ Antibodies |
| ☒ | ☐ Eukaryotic cell lines |
| ☒ | ☐ Palaeontology and archaeology |
| ☒ | ☐ Animals and other organisms |
| ☒ | ☐ Human research participants |
| ☒ | ☐ Clinical data |
| ☒ | ☐ Dual use research of concern |

## Methods

| n/a | Involved in the study |
|---|---|
| ☒ | ☐ ChIP-seq |
| ☒ | ☐ Flow cytometry |
| ☒ | ☐ MRI-based neuroimaging |
